# Dietary fibers inhibit obesity in mice, but host responses in the cecum and liver appear unrelated to fiber-specific changes in cecal bacterial taxonomic composition

**DOI:** 10.1038/s41598-018-34081-8

**Published:** 2018-10-22

**Authors:** Janice E. Drew, Nicole Reichardt, Lynda M. Williams, Claus-Dieter Mayer, Alan W. Walker, Andrew J. Farquharson, Stavroula Kastora, Freda Farquharson, Graeme Milligan, Douglas J. Morrison, Tom Preston, Harry J. Flint, Petra Louis

**Affiliations:** 1 The Rowett Institute, University of Aberdeen, Foresterhill, Aberdeen, AB25 2ZD UK; 2 Biomathematics and Statistics Scotland, University of Aberdeen, Foresterhill, Aberdeen, AB25 2ZD UK; 3 Aberdeen Fungal Group, University of Aberdeen, Foresterhill, Aberdeen, AB25 2ZD UK; 4 Institute of Molecular, Cell and Systems Biology, College of Medical, Veterinary and Life Sciences, University of Glasgow, Glasgow, G12 8QQ UK; 5 Scottish Universities Environmental Research Centre, University of Glasgow, Rankine Avenue, East Kilbride, G75 0QF UK

## Abstract

Dietary fibers (DF) can prevent obesity in rodents fed a high-fat diet (HFD). Their mode of action is not fully elucidated, but the gut microbiota have been implicated. This study aimed to identify the effects of seven dietary fibers (barley beta-glucan, apple pectin, inulin, inulin acetate ester, inulin propionate ester, inulin butyrate ester or a combination of inulin propionate ester and inulin butyrate ester) effective in preventing diet-induced obesity and links to differences in cecal bacteria and host gene expression. Mice (n = 12) were fed either a low-fat diet (LFD), HFD or a HFD supplemented with the DFs, barley beta-glucan, apple pectin, inulin, inulin acetate ester, inulin propionate ester, inulin butyrate ester or a combination of inulin propionate ester and inulin butyrate ester for 8 weeks. Cecal bacteria were determined by Illumina MiSeq sequencing of 16S rRNA gene amplicons. Host responses, body composition, metabolic markers and gene transcription (cecum and liver) were assessed post intervention. HFD mice showed increased adiposity, while all of the DFs prevented weight gain. DF specific differences in cecal bacteria were observed. Results indicate that diverse DFs prevent weight gain on a HFD, despite giving rise to different cecal bacteria profiles. Conversely, common host responses to dietary fiber observed are predicted to be important in improving barrier function and genome stability in the gut, maintaining energy homeostasis and reducing HFD induced inflammatory responses in the liver.

## Introduction

The intestinal microbiota interacts with host physiology via numerous putative metabolic and regulatory routes to impact health and disease^1,2^. The microbiota is implicated in the development of diet-induced obesity, but the underlying mechanisms are not fully elucidated^3,4^.

Dietary fibers are protective against the development of diet–induced obesity^5–7^. The effects of dietary fibers are largely attributed to their fermentation by gut bacteria and the uptake of metabolites, such as short chain fatty acids (SCFAs), by the colon epithelium, splanchnic tissues and peripheral organs. For example, butyrate stimulates intestinal gluconeogenesis from propionate and SCFA metabolism in the liver regulates glucose and lipid metabolism and energy homeostasis^8,9^. Dietary fiber and SCFAs are reported to limit body weight gain due to a reduction in food intake in rodents^10–12^ and are inversely associated with body weight gain in humans^13–15^.

Dietary fibers are diverse polysaccharides and oligosaccharides of plant origin. Gut bacteria vary in their ability to degrade and utilize dietary fiber and consequently different dietary dietary fibers profoundly alter the composition of the gut microbiota^16^. Our study aimed to firstly establish whether seven different dietary fibers (barley beta-glucan, apple pectin, inulin, inulin acetate ester, inulin propionate ester, inulin butyrate ester or a combination of inulin propionate ester and inulin butyrate ester), selected following *in vitro* fermentation studies^17^, were all equally protective in preventing diet-induced obesity in mice and whether this is related to specific cecal bacterial profiles. Since one potential mechanism for the action of dietary fibers is via SCFA production we also included inulin SCFA esters to assess the role of individual SCFA^18,19^. A second aim was to determine associated dietary fiber altered regulation of gene expression in the cecum, the first organ impacted by differences in the cecal bacteria, and the liver, the gatekeeper organ between the gut and the systemic circulation and whether this could be linked to cecal bacterial profiles.

To do this we used a well-defined model of diet-induced obesity. We have previously shown that 12 week old (24–25 grams body weight) C57Bl/6J male mice rapidly and predictably gain bodyweight, adiposity and liver lipid content when fed a HFD^20^. Using this model for the current study we manipulated dietary carbohydrate to replace a proportion of corn starch and cellulose in the HFD with different fermentable dietary fibers (barley beta-glucan, apple pectin, inulin, inulin acetate ester, inulin propionate ester, inulin butyrate ester or a combination of inulin propionate ester and inulin butyrate ester). We measured food intake and adiposity at the same time as analysing post-intervention profiles of bacteria collected from the cecal contents using Illumina MiSeq sequencing and associated host gene expression in the cecum and liver.

## Results

### Body composition and food intake

**Figure 1:**
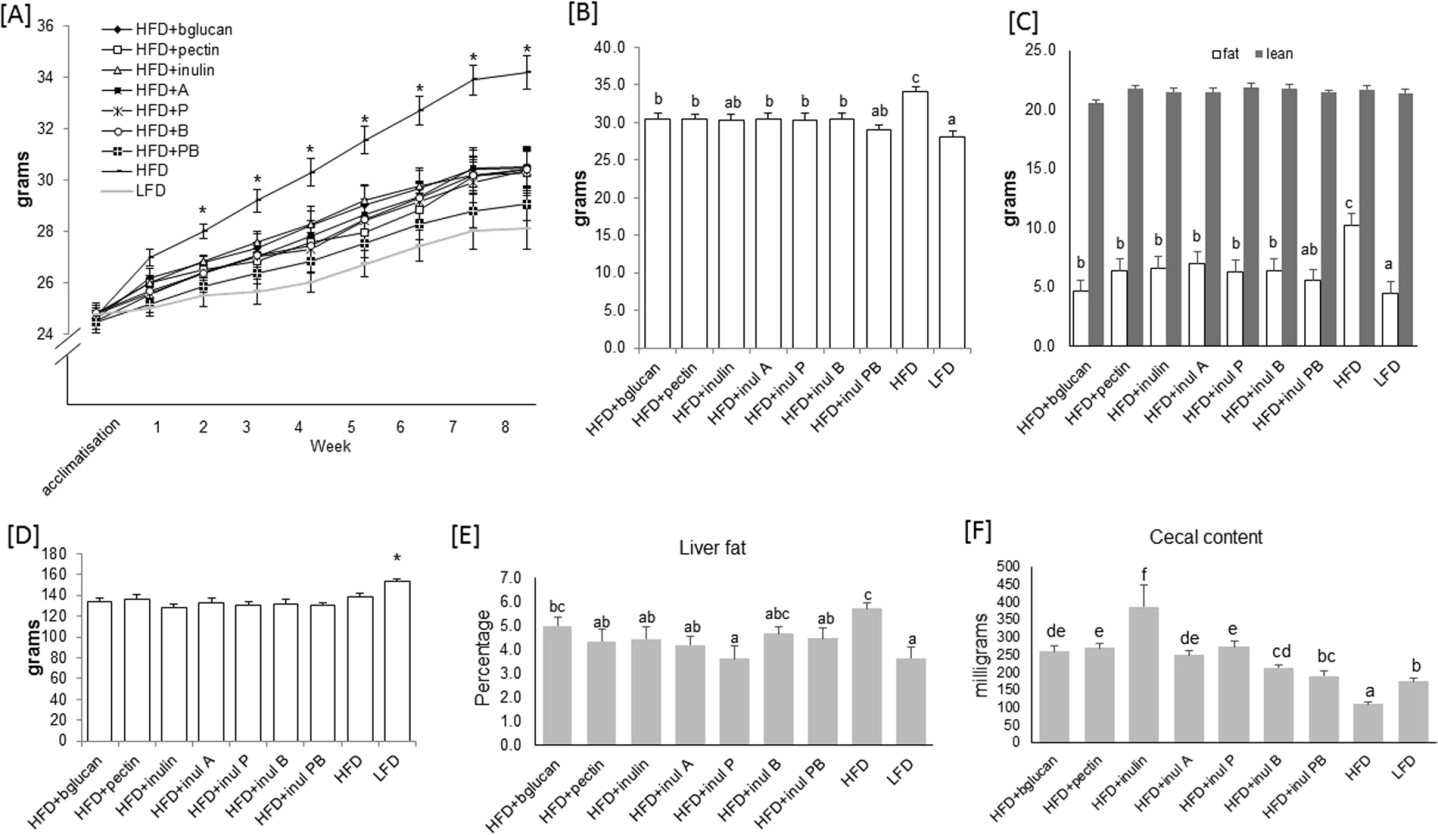
Mouse body weight, composition, cumulative food intake and cecal content (n = 12). (**A**) Body weight accumulation of the high fat diet (HFD) fed mice differed significantly from those of the HFD where 10% of the carbohydrate by weight (5% corn starch, 5% cellulose) was replaced by the following dietary fibers: beta glucan (HFD + bglucan), apple pectin (HFD + pectin), inulin (HFD + inulin), inulin acetate ester (HFD + inul A), inulin propionate ester (HFD + inul P), inulin butyrate ester (HFD + inul B), inulin propionate and butyrate ester, 5% each (HFD + inul PB) and low fat diet (LFD) fed mice from 2 weeks onwards until the end of the experiment. (**B**) Body weight of HFD fed mice at week 8 was significantly greater than mice consuming HFD + DF or LFD. Bodyweight of mice at week 8 fed HFD + DF was closer to that of LFD fed mice. (**C**) The increase in body weight seen in (**A**,**B**) is attributed to fat mass, which was increased in the HFD fed mice, while lean mass did not significantly differ with the dietary interventions as measured in mice at week 8. (**D**) Cumulative food intake measured over the course of the study did not differ in mice receiving either a HFD or HFD + DF. Mice consuming LFD consumed significantly more food. (**E**) Liver fat (**F**) Cecal content. Significant (p < 0.05) differences assessed by ANOVA with Fisher’s correction are indicated using lower case letters to distinguish differences between the diets.

Body composition and food intake of male C57BL/6 mice fed either HFD (high fat diet) (45% of energy from fat) (D12451), LFD (low fat diet) (10% fat by energy) (D12450B), or the HFD where 10% of the carbohydrate by weight (5% corn starch, 5% cellulose) was replaced by the following dietary fibers: beta glucan (HFD + bglucan) (Glucagel, DKSH, Milan Italy), pectin (HFD + pectin) (Sigma-Aldrich, Gillingham, UK), inulin (Beneo Orafti® HP, DKSH, London UK), inulin acetate ester (HFD + inul A), inulin propionate ester (HFD + inul P), inulin butyrate ester (HFD + inul B) or 5% each of inulin propionate and inulin butyrate ester (HFD + inul PB) (details of diets are provided in Supplementary File S1) for 8 weeks was assessed. HFD fed mice were heavier (*P* = 0.003) after 1 week (26.96 ± 0.32 g) compared to LFD (25.01 ± 0.29 g) and HFD + DFs. HFD mice remained heavier compared to other diet groups throughout the experiment (Fig. 1A,B). HFD mice had a greater fat mass at 8 weeks (Fig. 1C). There were no differences in lean mass in any of the diet groups (Fig. 1C). Colon length, weight/cm ratios and liver weights were measured and were not found to be significantly different (data not shown). Food intake was not different in mice fed HFD (138.22 ± 3.54 g) or HFD + DF (Fig. 1D). LFD fed mice had a higher food intake by weight compared to other diets (153.36 ± 2.18 g) (Fig. 1D).

Total liver lipid was lower in mice fed LFD and all HFD + DFs (Fig. 1E), but did not reach significance for HFD + bglucan and HFD + inul B *vs*. HFD (Fig. 1E). Cecum content weight varied with diet (*P* < 0.001) (Fig. 1F). HFD fed mice had the lowest cecal content weight (110.0 ± 5.4 mg) (Fig. 1F), while HFD + inulin yielded the highest weight (Fig. 1F).

### Circulating hormones and inflammatory markers

**Figure 2:**
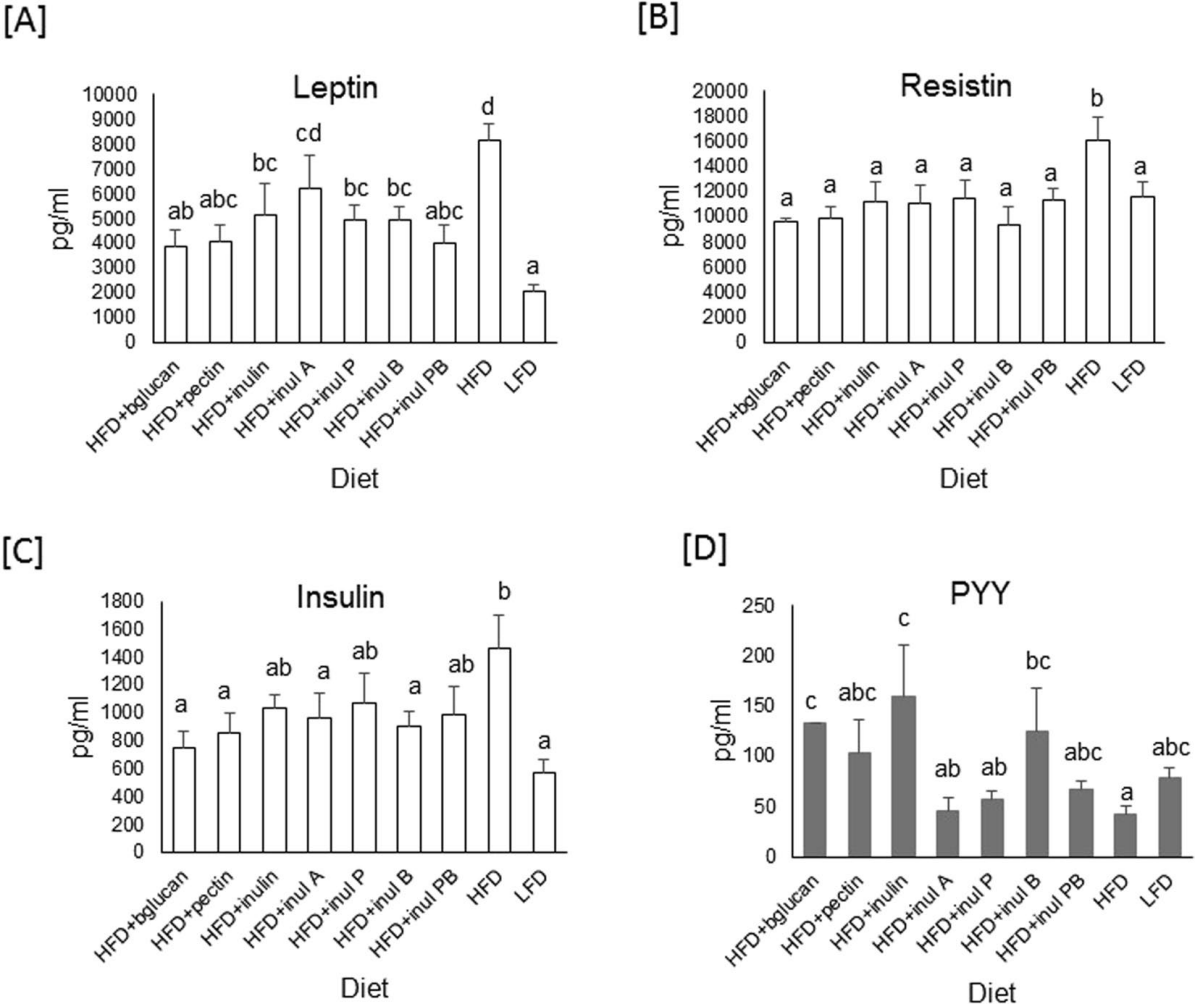
Plasma leptin, insulin, resistin and PYY responses in mice fed a high fat diet (HFD), HFD where 10% of the carbohydrate by weight (5% corn starch, 5% cellulose) was replaced by beta glucan (HFD + bglucan), apple pectin (HFD + pectin), inulin (HFD + inulin), inulin acetate ester (HFD + inul A), inulin propionate ester (HFD + inul P), inulin butyrate ester (HFD + inul B), inulin propionate and butyrate ester, 5% each (HFD + inul PB) and low fat diet (LFD). Mean (**A**) Leptin, (**B**) Insulin, (**C**) Resistin (n = 5–7) and (**D**) PYY (n = 3–7). Significant (p < 0.05) differences assessed by ANOVA with Fisher’s correction are indicated using lower case letters to distinguish differences between the diets.

Circulating leptin, resistin and insulin levels measured in plasma from cardiac puncture were lower in HFD + DFs and LFD mice (Fig. 2A–C). Gut hormones, PYY, GIP and ghrelin were measured in hepatic portal vein. Plasma PYY (p = 0.041) was higher in HFD + bglucan, HFD + inulin and HFD + inul B ester *vs*. HFD mice (Fig. 2D). There were no differences in GIP or ghrelin (data not shown). GLP-1 was undetectable.

### Cecal bacteria

**Figure 3:**
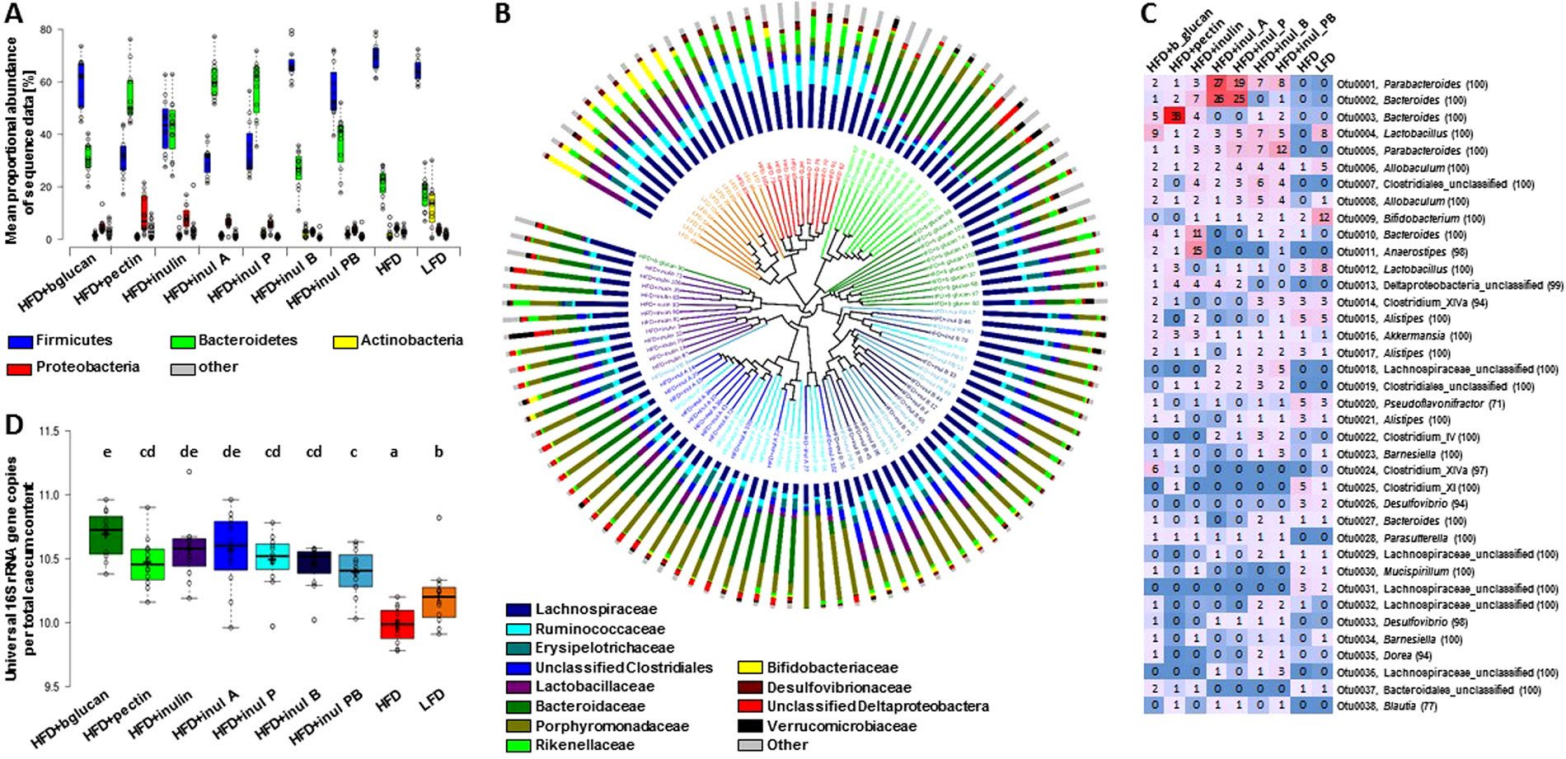
Cecal bacterial composition. (**A**) Mean proportional abundance of bacterial sequencing data at phylum level (n = 12). Centre lines show the medians and box plot limits indicate the 25^th^ and 75^th^ percentiles as determined by R software; whiskers extend 1.5 times the interquartile range from the 25^th^ and 75^th^ percentiles, individual animals are presented by dots. (**B**) Bray Curtis cluster dendrogram of bacterial composition at family level in mice fed mice fed a high fat diet (HFD, red), HFD where 10% of the carbohydrate by weight (5% corn starch, 5% cellulose) was replaced by beta glucan (HFD + bglucan, dark green), apple pectin (HFD + pectin, light green), inulin (HFD + inulin, purple), inulin acetate ester (HFD + inul A, blue), inulin propionate ester (HFD + inul P, light blue), inulin butyrate ester (HFD + inul B, dark blue), inulin propionate and butyrate ester, 5% each (HFD + inul PB, blue-green) and low fat diet (LFD, orange). Also see high resolution of Fig. 3B in Supplementary File S3 (**C**) Heat map of relative abundance (expressed as average percentage to total sequences per diet) of the 38 most abundant OTUs (≥0.5% of total sequences; data for all OTUs are given in Supplementary File S4). (**D**) Total universal 16S rRNA gene copies in cecal content. Centre lines show the medians and box plot limits indicate the 25^th^ and 75^th^ percentiles as determined by R software; whiskers extend 1.5 times the interquartile range from the 25^th^ and 75^th^ percentiles, individual animals are presented by dots.

Illumina MiSeq sequencing of 16S rRNA gene amplicons derived from cecal contents revealed a strong influence of dietary fiber supplementation on bacterial composition. Phylum-level analysis showed that Firmicutes were dominant in HFD + bglucan, HFD + inul B, HFD + inul PB, HFD and LFD groups, whereas Bacteroidetes were the most proportionally abundant phylum in the HFD + pectin, HFD + inul A and HFD + inul P groups (Fig. 3A). HFD + inulin had equal proportions of each of the two phyla (Fig. 3A). HFD + pectin and HFD + inulin had the highest percentage levels of Proteobacteria (mostly Deltaproteobacteria), but there was large individual variation (Fig. 3A). Mice fed the LFD had higher proportions of Actinobacteria belonging to the *Bifidobacteriaceae* family relative to those fed HFD diets with or without added dietary fibers (Fig. 3A,B). Large differences were also observed at family (Fig. 3B, Supplementary File S3) and Operational Taxonomic Unit (OTU) level (Fig. 3C, Supplementary File S4) between different groups, some of which were associated with specific diets (Supplementary File S4), including Ruminococcaceae and Lachnospiraceae in the HFD group, Bacteroidaceae in the HFD + pectin group and Porphyromonadaceae in the HFD + inulin acetate ester group (Fig. 3B). The 38 most abundant OTUs (≥0.5% of total sequences; data for all OTUs are given in Supplementary File S4) are shown in the heat map of relative abundance (expressed as average percentage to total sequences per diet) (Fig. 3C).

Cecum bacterial profiles clustered separately from one another based upon diet for most animals. LFD and HFD with no added fiber clustered together, indicating that presence of fiber is a major driver of overall community composition. Metastats and LEfSe (Linear discriminant analysis effect size) analyses confirmed that there were a large number of significant differences in constituent taxa between these two groups and all the fiber-containing groups together (Supplementary File S4). In contrast, the two dietary groups with no added fibers (LFD and HFD) showed fewer significant differences between them (Supplementary File S5). We also observed that the four inulin esters separated into two clear groups, with HFD + inul A/HFD + inul P and HFD + inul B/HFD + inul PB clustering together (Fig. 3B, Supplementary File S3). Two different clustering methods, (Jaccard, which incorporates only presence/absence of OTUs, and Bray Curtis, which also incorporates proportional abundances of each OTU when comparing dissimilarities) revealed a highly significant effect of diet (*P* < 0.001). Pairwise comparison of all dietary groups showed that clustering in both trees was significant (*P* < 0.001) with the exception of HFD + inul A compared to HFD + inul P (*P* = 0.018 Jaccard; *P* = 0.071 Bray Curtis) and HFD + inul B compared to HFD + inul PB (not significant). AMOVA analysis essentially resulted in the same outcome (HFD + inul A compared to HFD + inul P *P* < 0.001 Jaccard; *P* = 0.058 Bray Curtis; HFD + inul B compared to HFD + inul PB not significant; all other comparisons *P* < 0.001). LEfSe analysis including all individual dietary groups revealed the highest number of significant associations for the HFD fed group, followed by HFD + pectin (Supplementary File S4). Thus there were no consistent changes in phylogenetic (16S rRNA-based) community composition with weight gain and fiber intake on the HFD diets, despite the significant impacts of individual fibers on bacterial composition when incorporated in the HFD. However, the possibility of common changes in some functional microbial group/groups that impact on weight gain, or in some low-abundance taxon that has a major effect on physiology cannot be ruled out.

Bacterial diversity was higher in HFD fed mice compared to mice on all other diets and there were also significant differences in diversity indices between different diets incorporating different dietary fibers that were consistent with the Bray-Curtis clustering (Supplementary File S5). All diets containing dietary fibers had higher total bacterial 16S rRNA gene copies compared to either HFD or LFD, indicating greater bacterial numbers per g of cecal contents. HFD fed mice had the lowest bacterial abundance (Fig. 3D).

### Whole genome modulation in liver and cecum in response to dietary fibers

Global microarray analysis was conducted on liver and cecum of mice (n = 6) from HFD, LFD, HFD + inul and HFD + inul PB groups. The HFD + inul PB were chosen as the lowest weight gain and analysis of the HFD + inulin group allowed identification of any inulin *vs*. inulin ester effects. Comparison with HFD and LFD mice distinguished the effects of dietary fibers from body weight/adiposity effects.

Principal component analysis (PCA) of normalised microarray data indicated diet associated cecum and liver gene expression, explaining 22.99% and 19.22% of the variation in the data respectively (Supplementary File S6 Fig. S2A,B). PCA analysis of cecal gene expression indicated a tendency for the HFD + inul and HFD + inul PB fed mice to cluster together distinct from the HFD and LFD fed mice (Supplementary File S6 Fig. S2A). However, PCA analysis of liver gene expression profiles did not reveal distinct clusters (Supplementary File S6 Fig. S2B).

Subsequent analysis was applied to identify probe IDs indicating a > 1.5 fold difference (*P* < 0.01) in gene expression when compared to HFD mice. Using the cut off set at > 1.5 fold difference (*P* < 0.01), 741 probe IDs in HFD + inulin, 1614 in HFD + inul PB ester and 151 in LFD fed mice showed differences in expression levels compared to HFD fed mice (Supplementary File S6 Fig. S2C–E, GEO Accession no. GSE106375). In liver, there were 68 probe IDs in HFD + inulin, 53 in HFD + inul PB and 196 in LFD mice (GEO Accession no. GSE106375) showing differences in gene expression compared to HFD mice. Greater numbers of probe IDs were identified in the cecum of mice consuming HFD + DFs compared to LFD, indicating that differences in gene expression are greater with DF supplementation, rather than simply altered in association with body weight/adiposity. The same selection criteria identified a greater number of differences in gene expression regulated by LFD in liver compared to HFD supplemented with DFs.

### Validation of selected gene targets in cecum and liver and regulation in response to dietary fibers

Validation of microarray data was conducted to confirm altered regulation of selected gene targets in response to HFD + inulin and HFD + inul PB. Comparison of the selected gene targets was assessed in the microarrayed samples and also in response to the other diet interventions using a custom designed RT Profiler PCR Array of cecum and Taqman assays of cecum and liver.

**Figure 4:**
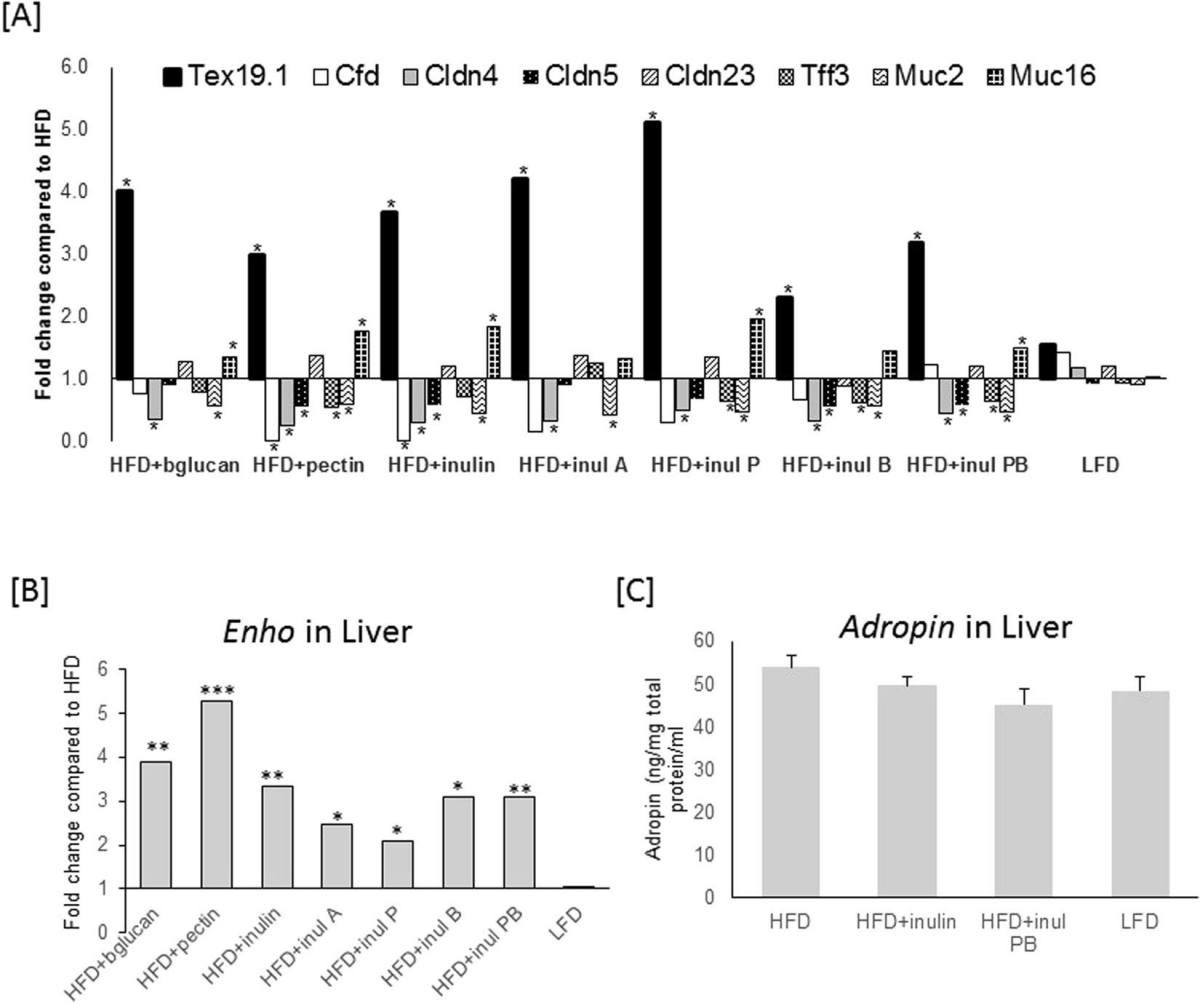
Validation of microarray data and responses in mice fed a high fat diet (HFD), where 10% of the carbohydrate by weight (5% corn starch, 5% cellulose) was replaced by beta glucan (HFD + bglucan), apple pectin (HFD + pectin), inulin (HFD + inulin), inulin acetate ester (HFD + inul A), inulin propionate ester (HFD + inul P), inulin butyrate ester (HFD + inul B), inulin propionate and butyrate ester, 5% each (HFD + PB) and low fat diet (LFD). (**A**) RT Profiler PCR Array of selected gene targets showing altered gene regulation in response to HFD + inul and HFD + PB from microarray data analysis of cecum. Fold change was calculated relative to HFD fed mice using mean gene target normalised to *UBE2D2* (n = 6). (**B**) *Enho* gene expression in liver of mice fed HFD + DF or LFD relative to HFD. Fold change was calculated relative to HFD fed mice using mean *Enho* normalised to *UBE2D2* (n = 6). (**C**) Adropin levels in liver. A Student’s t-test based on delta CT values was applied to test comparisons with HFD fed mice. ^*^P < 0.05, ^**^P < 0.01, ^***^P < 0.001.

Genes were selected on the basis of large differences in response to consumption of HFD + inulin or HFD + inul PB ester diets, and involvement in gut barrier function in the case of cecum microarray targets. RT Profiler PCR Array confirmed that cecal genes were associated with dietary fiber supplementation rather than adiposity, with no differences observed in target gene expression in LFD fed mice (Fig. 4A). Selected gene targets were regulated similarly in response to all HFD + DF diets, including higher levels of cecal *Tex19*.*1* (Testis expressed 19.1) (Fig. 4A) and *Muc16* (Mucin 16), except HFD + inul A and HFD + inul B (Fig. 4A). Higher *Cldn23* (Claudin 23) expression was not confirmed by RT Profiler PCR.

Significant reduced levels of cecal *Cldn4* (Claudin 4) and *Muc2* (Mucin 2) were confirmed for all HFD + DFs (Fig. 4A), while *Cfd* (Complement Factor D) down regulation observed in the majority of HFD + DFs failed to reach significance (Fig. 4A). Lower *Cldn5* (Claudin 5) expression was observed in HFD + pectin, HFD + inulin, HFD + inul B and HFD + inul PB (Fig. 4A). *Tff3* (Trefoil Factor 3) was lower in HFD + pectin, HFD + inul P, HFD + inul B and HFD + inul PB (Fig. 4A). In summary, the majority of microarray identified genes were similarly regulated by all diets containing dietary fibers (Fig. 4A).

Microarray analysis revealed increased *Enho* (Energy Homesostasis Associated) expression in liver for all HFD + DF diets relative to the HFD diet, with the greatest levels observed in response to HFD + inul PB. *Enho* expression in LFD mice compared to HFD fed mice was not altered (Fig. 4B) and levels of adropin, encoded by *Enho*, were not increased in liver (Fig. 4C).

### Molecular interaction networks and integration of gene responses to inulin and inulin propionate and butyrate esters in cecum and liver

**Figure 5:**
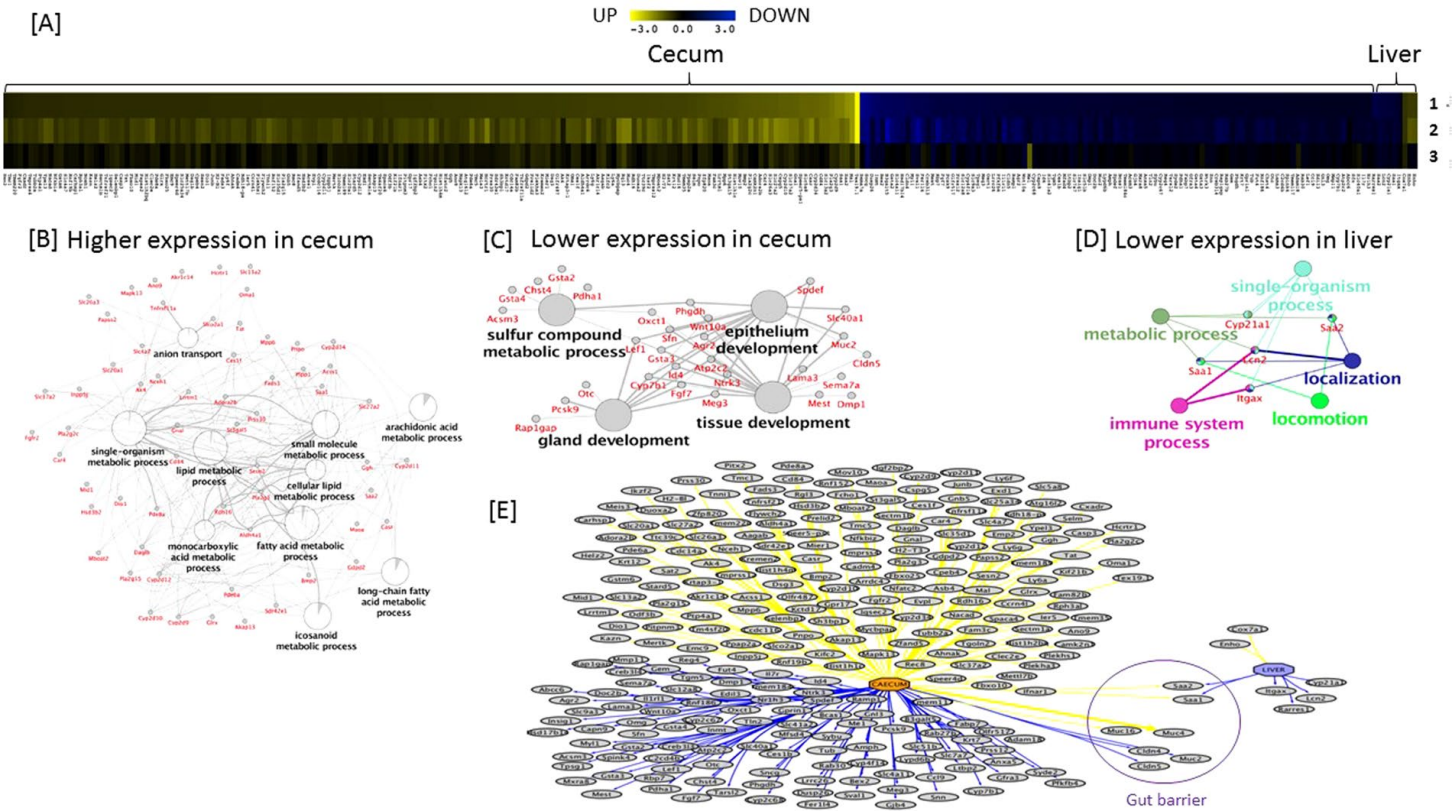
Altered ontologies and pathways associated with known genes showing 1.5 fold differences (*P* < 0.01) in expression in cecum and liver of mice fed a high fat diet (HFD) where 10% of the carbohydrate by weight (5% corn starch, 5% cellulose) was replaced by inulin (HFD + I), inulin propionate and butyrate ester, 5% each (Inulin PB) and low fat diet (LFD) relative to HFD fed mice. The networks are organized into significantly enriched GO categories. (**A**) Heat map illustrating up and down regulated genes. Cytoscape networks of up-regulated (**B**) and down-regulated genes in cecum (**C**) and liver (**D**). (**E**) Integrated analysis illustrating up- and down-regulated genes indicated by yellow and blue lines respectively in cecum and liver. Details of gene list shown in Supplementary File S8.

The validation of gene expression using RT Profiler PCR Array and Taqman assays revealed common responses to dietary fiber consumption in cecum (Fig. 4A) and liver (Fig. 4B) irrespective of the type of dietary fiber or dietary fiber specific differences in cecal bacterial composition (Fig. 3). Pathway analysis was carried out on a sub-set of normalised microarray gene expression data of known genes (predicted genes and unnamed transcripts were excluded) that showed >1.5 fold differences *P* ≤ 0.01 in gene expression compared to HFD in cecum and liver in response to HFD + inul and HFD + inul PB, but not LFD. This gene sub-set consisted of 168 genes with higher and 102 with lower levels of gene expression in cecum and 2 with higher and 6 with lower levels of gene expression in liver (Supplementary Files S7 and S8) when compared to HFD fed mice. Common transcriptional responses to HFD + inul and HFD + inul PB are shown (Fig. 5A). A Cytoscape network of genes showing common transcriptional responses to HFD + inul and HFD + inul PB illustrates the categories these genes fall into, including anion transport, lipid, small molecule, single-organism, long-chain fatty acid, cellular lipid, monocarboxylic, arachidonic, icosanoid and fatty acid metabolic processes being upregulated (Fig. 5A), while sulphur compound metabolic process epithelium, gland and tissue development were downregulated in the cecum (Fig. 5B). GO (Gene Ontology) terms enriched in liver in response to HFD + inul and HFD + inul PB were localisation, locomotion, immune system, metabolic and single-organism processes (Fig. 5C).

**Figure 6:**
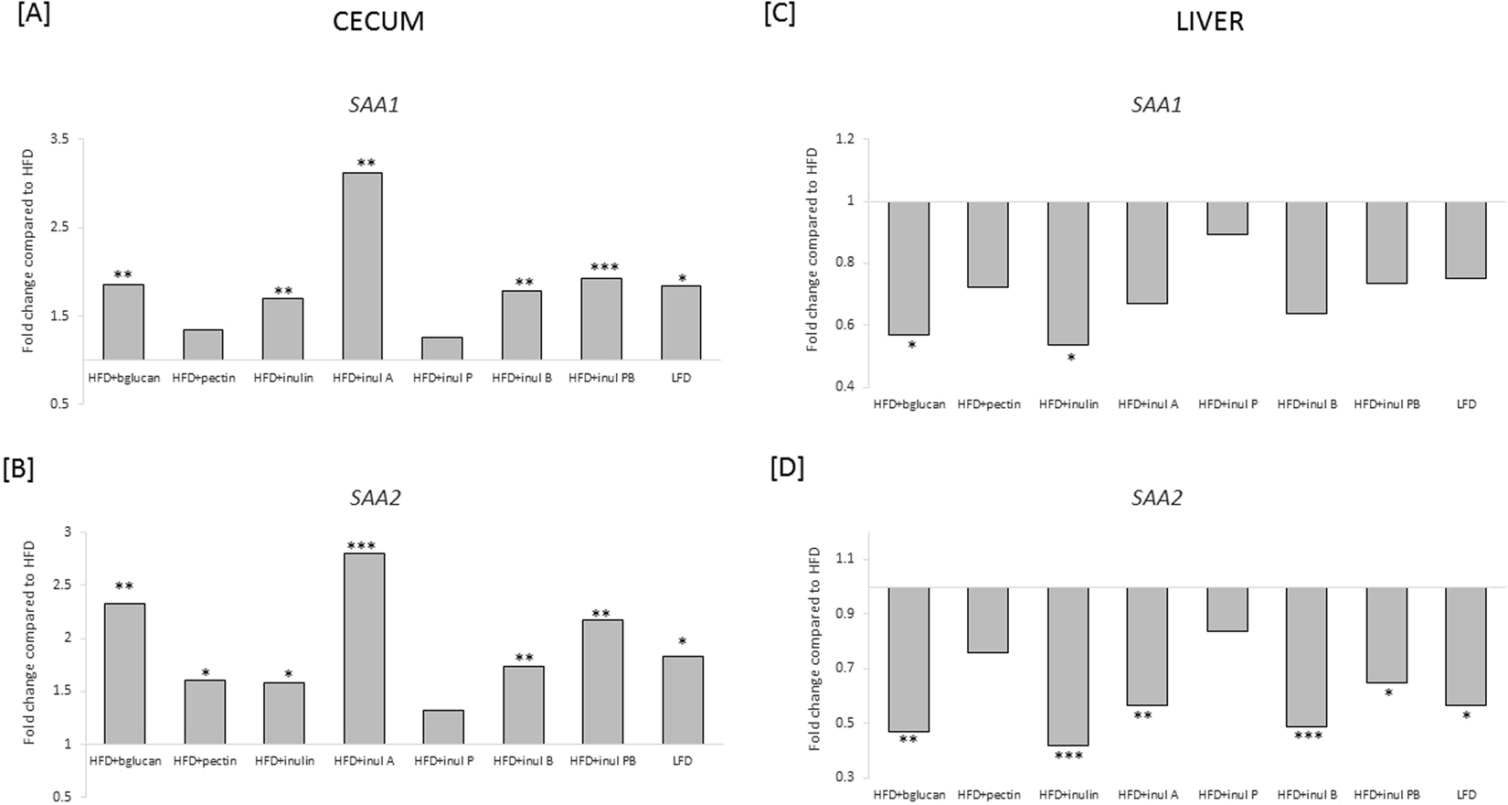
Gene expression of *SAA1* and *SAA2* relative to high fat (HFD) fed mice in cecum and liver in mice fed a HFD where 10% of the carbohydrate by weight (5% corn starch, 5% cellulose) was replaced by beta glucan (HFD + bglucan), apple pectin (HFD + pectin), inulin (HFD + inulin), inulin acetate ester (HFD + inul A), inulin propionate ester (HFD + inul P), inulin butyrate ester (HFD + inul B), inulin propionate and butyrate ester, 5% each (HFD + PB) and low fat diet (LFD). Gene expression was calculated relative to HFD fed mice using mean gene target normalised to *UBE2D2* (n = 5–6). A Student’s ttest was applied to test comparisons with HFD fed mice. ^*^P < 0.05, ^**^P < 0.01, ^***^P < 0.001.

The Cytoscape network revealed *SAA1* (Serum Amyloid A1) and *SAA2* (Serum Amyloid A2), involved in inflammatory responses, differed in response to consumption of HFD + inul and HFD + inul PB esters in both cecum (higher) and liver (lower) (Fig. 5E) compared to HFD. The differences in gene expression were confirmed by real-time PCR (Fig. 6). However, higher *SAA1* levels in cecum failed to reach significance for HFD + pectin and HFD + inul P, as did *SAA2* for HFD + inul P (Fig. 6A,B). Lower levels of *SAA1* in liver were observed in response to consumption of HFD + DFs, reaching significance in HFD + bglucan and HFD + inulin fed mice. While the lower level of *SAA2* in liver was significant for all HFD + DFs, except from HFD + pectin and HFD + inul P. LFD mice also showed comparable differences in *SAA1* and *SAA2* in cecum and liver measured using real time PCR (these differences were noted in microarray analysis, but had failed to meet the significance cut off) (*P* < 0.01) (Fig. 6).

## Discussion

This study reports comprehensive analysis of seven dietary fibers combined with HFD on cecal bacterial profiles and host gene expression in the cecum and liver of mice. While there were fiber-specific differences in cecal bacterial composition, all dietary fibers tested prevented obesity and yielded similar responses in body composition and host gene expression in cecum and liver of a number of gene targets identified by microarray. The responses to dietary fiber of the gene targets selected for further analysis confirmed a similar outcome, implying that while cecal bacterial profiles differ specific to each dietary fiber, this results in collective outcomes in the expression of certain host genes. Despite the differences in bacterial profiles associated with specific fibers we established common gene expression differences in the host irrespective of which fiber was incorporated in the HFD. This finding was a significant outcome of our study and implies that bacterial composition *per se* may not be causal in protecting against HFD-induced weight gain. However, there is a possibility that common changes in microbial groups producing particular metabolites or signalling molecules, or a low-abundance taxon that induces major effects on physiology, could be contributing to the observed effects on weight gain and metabolism.

Nonetheless, replacement of dietary starch by dietary fibers in these defined diets was predicted to decrease the supply of carbohydrate-derived calories in the upper GI tract by 12.4%. Calories arising from bacterial fermentation of dietary fibers cannot be calculated exactly, but iso-caloric replacement is based on the assumption that dietary fibers provide 50% of the calorific value of digestible carbohydrates^21^. The cellulose incorporated in the diets (International Fiber Corporation) has no calorific value for the host and was replaced with dietary fibers in the HFD + DF diets. Given that there was no detectable change in cumulative food intake (Fig. 1D) between the HFD and the HFD diets incorporating dietary fiber, together with protection against diet-induced obesity (Fig. 1A–C), our results indicate that the net calorie gain may have been lower than this.

While composition of the cecal bacteria were different between the different dietary fibers, numbers of cecal bacteria per g cecal contents were enhanced by all dietary fibers. It is known that gut bacterial composition is affected by the addition of dietary fibers to the diet^16,17,22^, but also by dietary fat and protein^23^, by gut turnover/transit time and the gut environment (e.g. pH)^2^. In contrast to earlier reports^24,25^ we found little difference between the HFD and LFD diets in the representation of Bacteroidetes and Firmicutes phyla, but Actinobacteria were proportionally less abundant in HFD mice, except for HFD + pectin and HFD + inulin. Major differences in cecal Bacteroidetes and Firmicutes proportions were seen between the HFD + DFs. For the three non-esterified dietary fiber diets, Bacteroidetes were proportionally favoured on HFD + pectin and Firmicutes on HFD + bglucan, while the two phyla were approximately equally represented on HFD + inulin. These differences appear largely due to dietary fiber specific responses at the OTU level (Supplementary File S4)^16^. There were also major differences in the proportions of these two phyla and at the OTU level with the four esterified inulin substrates. It has been reported that SCFA differentially affect the growth of members of these two phyla through pH-dependent stress^26^. However, there is no evidence that these major differences in cecal bacterial composition differentially affect adiposity apart from decreased weight gain on HFD.

There was no significant impact of dietary fibers in GIP or ghrelin (GLP-1 levels were undetectable). However, levels of PYY, leptin, insulin and resistin differed in HFD + DF compared to HFD fed mice. There were no consistent patterns observed in observed significant differences with consumption of the different fibers. Leptin levels were significantly higher in mice fed HFD + inulinA compared to HFD + bglucan only. While there was some indication that HFD + bglucan had significantly higher levels of PYY compared to HFD + inulinA and HFD + inulinP. However, notably PYY plasma levels were observed to be variable in mice. While there was a tendency for increased PYY this was only significantly increased in HFD + bglucan, HFD + inul and HFD + inulin B fed mice (Fig. 2D). This is in contrast to reports which have reported SCFA upregulation of PYY^4^. However, there were no differences in cumulative food intake between HFD and HFD + DF mice (Fig. 1D). Likewise there was no transcriptional response of *FFAR3* in cecum (measured by microarray analysis) in response to dietary fibers (GEO Accession no. GSE106375), while *FFAR2* was only down-regulated in HFD + inul + PB mice. This is not definitive evidence, but may indicate that these pathways are not a major influence on the effects of dietary fiber on adiposity. Levels of leptin, insulin and resistin are directly associated with adiposity and HFD + DF mice with lower adiposity consequently have lower levels of these hormones.

In contrast to the diet specific differences in cecal bacterial profiles, the transcriptional responses in cecum and liver are similar. Nonetheless, there were instances of differences in expression of selected gene targets associated with individual fiber diets, but these did not show any consistent pattern that would permit speculation on potential physiological outcomes. Microarray analysis indicated that many genes and pathways modulated in response to dietary fibers are epithelial, with a number involved in gut barrier function (Fig. 5). The gut barrier protects against ingress of harmful agents while allowing nutrient absorption. The myriad genes generating the proteins required to maintain healthy gut barrier function is still not fully understood. However, there are indications that the observed changes in gut barrier gene expression in the current study are favourable to improved barrier function. The altered expression of claudins have potential to alter trans-epithelial and strand tightness of tight junctions^27^. Mucins are another complex group of molecules known to be important components of the gut barrier with *Muc2* deficiency, consistently expressed at lower levels in fiber fed mice in our study, reported to protect mice from diet-induced fatty liver disease and obesity^28^. GO also indicated higher levels of anion transport genes, including *SLC* transporters which are widely expressed in epithelia, particularly associated with barrier function^29^. Both *SLC5A8* and *SLC26A3*, were elevated in cecum (Fig. 5/Supplementary File S7, GEO Accession no. GSE106375) in response to HFD + inul and HFD + inul PB, and are tumour suppressors^30,31^ with *SLC5A8* also linked to butyrate and propionate uptake^32^. These results, together with the associated evidence of reduced inflammation in the liver, leads us to conclude that observed changes in gut epithelial genes favour an improvement in gut barrier function.

*SAA1* and *SAA2*, the main acute phase isoforms of serum amyloid A, are expressed by the lumenal surface epithelium lining the colon^32^ and are thought to provide an anti-bacterial role, assisting in maintenance of epithelial immune homeostasis^32^. The encoded proteins are secreted into the lumen, play a role in innate recognition of Gram-negative bacteria, reduce bacterial viability^32,33^ and are linked to reduced risk of inflammatory bowel disease^32^. *SAA1* and *SAA2* were downregulated in liver in response to HFD + DF. HFD increases the inflammatory response in liver with an increase in circulating SAA^20^. The reduced inflammatory response in liver is evidenced by lower levels of *SAA1* and *SAA2*. Studies have shown that damaged gut epithelium results in elevated levels of circulating SAA most likely derived from liver^34^. Thus, the opposing effects of dietary fibers on *SAA1* and *SAA2* in cecum and liver may be linked (Fig. 5E).

Dietary fibers upregulate *Tex19*.*1* which has restricted expression in pluripotent stem cells^35^ and inhibits retrotransposons^36^. *Tex19*.*1* potentially stabilises the gut stem cell genome during replication and renewal of the gut epithelium, linking dietary fiber intake to anticancer effects.

There was a smaller subset of gene changes in common in the response to consumption of dietary fibers in the liver compared to those identified in the cecum measured by microarray analysis. Enho was chosen for further analysis following reports that its encoded protein, adropin, is a hormone involved in energy homeostasis and lipid metabolism, with adropin deficiency associated with obesity and insulin resistance^36^. The higher level of Enho expression in liver is seen with all dietary fibers tested and may be an important factor in reducing adiposity. Nonetheless, this was not reflected in higher levels of liver adropin, the protein encoded by Enho. It has been reported that Enho expression in liver results in increased circulating adropin^37^ and it may be that the elevated Enho expression in the liver may produce increased adropin secretion while liver adropin levels remain stable (Fig. 4C). Adropin is involved in energy homeostasis and lipid metabolism, with deficiency associated with obesity and insulin resistance^36^. Treatment with synthetic adropin reduces weight gain^36,38^ in agreement with prevention of weight gain in HFD + DF mice showing higher levels of liver Enho when compared to HFD fed mice. However, Enho gene expression was not altered in HFD compared to LFD mice, indicating that the response was a consequence of dietary fiber intake. Supporting our findings, LFD mice were reported to have reduced levels of adropin compared to mice fed chow, which is a rich source of fiber^36^. The consequences of increased *Enho* expression specifically in the liver may form the basis for explaining the beneficial effects of dietary fibers on metabolic health. Despite high levels of *Enho* expression in cecum (detected by microarray) it is not differentially expressed in response to the supplementation of the HFD with dietary fibers. It was noted that changes in other liver targets, such as Lcn2 (Lipocalin 2) and Itagx (integrin subunit alpha X), which are expressed at lower levels in the microarray analysis of HFD + inulin and HFD + inulPB compared to HFD provide further support for the protective effect of dietary fibers on liver. Lcn2 and Itagx are reported to be key inflammatory markers with activation of these markers indicative of metabolic and inflammatory stress in the liver^39–41^. This also further substantiates our contention that dietary fibers may improve barrier function and protect the liver from the inflammatory effects of consuming a HFD.

Our study provides a novel insight on the impact of dietary fiber on the cecal bacteria and host responses to diet-induced obesity, revealing that of the seven dietary fibers tested, all exert a similar effect on reducing adiposity and cecum and liver expression of the selected gene targets. It should be noted however, that consumption of fibers of different particle size have been shown to differentially affect metabolic and inflammatory responses in mice^42^. Dietary fiber-induced resistance to diet-induced obesity in this study is potentially mediated by the hormone adropin, as indicated by the liver specific increased levels of *Enho*. Additionally, improved gut barrier function, characterised by regulation of *Tex19*.*1* and altered mucins, claudins and epithelial solute transporters is associated with reduced expression of markers of inflammation and accumulation of fat in liver. The studies reported in the present paper were conducted in mice with further studies in humans needed to determine the effects of dietary fibers on modulating obesity. The already known associations of adropin and human health provide a useful focus for further study of translational potential to humans. In conclusion the effects of fiber consumption on a high fat diet has potential implications for health that are apparently not directly related to cecal bacterial community composition, although we cannot exclude the possibility that they may be related to total microbial populations and, or their overall metabolic activity.

## Methods

### Animals and dietary intervention

The animal studies were licensed under the Animal (Scientific Procedures) Act of 1986 and in accordance with the European Directive on the Protection of Animals used for Scientific Purposes 2010/63/E following ARRIVE guidelines and received approval from the Rowett Institute’s Ethical Review Committee. Male C57BL/6 mice, 12 weeks of age and 24–25 g in weight (Harlan, Bicester, UK), were randomly assigned to one of nine dietary groups (n = 12) and fed, either: **1**. HFD (high fat diet) (45% of energy from fat) (D12451) **2**. LFD (low fat diet) (10% fat by energy) (D12450B), or the HFD where 10% of the carbohydrate by weight (5% corn starch, 5% cellulose) was replaced by the following dietary fibers: **3**. beta glucan (HFD + bglucan) (Glucagel, DKSH, Milan Italy) **4**. pectin (HFD + pectin) (Sigma-Aldrich, Gillingham, UK) **5**. inulin (Beneo Orafti® HP, DKSH, London UK) **6**. inulin acetate ester (HFD + inul A) **7**. inulin propionate ester (HFD + inul P) **8**. inulin butyrate ester (HFD + inul B) **9**. 5% each of inulin propionate and inulin butyrate ester (HFD + inul PB) (details of diets are provided in Supplementary File S1) for 8 weeks. The cellulose used in the diets contributes zero calories. Further details of animals, dietary intervention and sample collection are provided in Supplementary File S2.

### Cecal bacterial analysis

Genomic DNA (gDNA) was extracted from the cecum contents using the FastDNA® SPIN Kit for Soil (MP Biomedicals, Illkirch, France). Total cecal bacterial abundance was estimated following the dietary interventions by quantitative PCR and the V3-V4 region of bacterial 16S rRNA genes were sequenced on the Illumina MiSeq using a v3 flow cell with 2 × 300 bp paired end reads (full details of the subsequent analysis steps used are available in Supplementary File S2). Sequencing data generated during this study are available in the SRA database under SRA accession SRP117745 (accessible at http://www.ncbi.nlm.nih.gov/sra/SRP117745).

### Plasma hormones

Hormones were measured in plasma from cardiac puncture (CP) or from hepatic portal vein (HPV) (n = 4–8) collected with incorporation of Protease Inhibitor Cocktail for General Use (Sigma cat# P2714) using Millipore’s MILLIPLEX MAP Mouse Metabolic Hormone Magnetic Bead panel (Merck Millipore, Feltham, UK).

### Liver fat

Total fat was extracted from left liver lobes based on the method by Folch *et al*.^43^ and weight of fat per microgram of liver tissue calculated.

### Whole genome microarray analysis

Total RNA extracted from liver and cecum using an RNeasy Mini Kit (Qiagen, Crawley, UK) was microarrayed with SurePrint G3 Mouse GE 8 × 60 K Microarray G4852A (Agilent Technologies, UK) (Supplementary File S2). Data are deposited in NCBI’s Gene Expression Omnibus^44^ and are accessible through GEO Series accession number GSE106375 (www.ncbi.nlm.nih.gov/geo/query/acc.cgi?acc=GSE106375). Details of statistical analysis of microarray data is provided in Supplementary File S2.

### Confirmation of microarray identified differences in gene expression using custom designed RT Profiler PCR arrays

Genes showing altered responses to HFD + inulin or HFD + inul PB esters in cecum were identified from microarray analysis and validated using a custom designed RT Profiler PCR Array (Qiagen) (Supplementary File S2).

### Real-time PCR

Complementary cDNA templates for real-time PCR assays were prepared from Superscript II (Invitrogen) reverse transcribed total RNA and Taqman assays were conducted with duplex target and reference gene *UBE2D2*. (Supplementary File S2).

### Liver adropin (Enho)

An enzyme-linked immunosorbent assay (ELISA) kit (Cusabio USA) was used to measure adropin in accordance with manufacturer’s instructions. (Supplementary File S2).

### Statistical analysis

Details of statistical analysis of cecal microbiota sequencing, cecum and liver microarray data can be found in Supplementary File S2. Other data are presented as mean ± SEM and analysed using GenStat (Gen Stat®13th Edition (VSN International, Ltd., Hemel Hempstead, UK) apart from the RT-PCR data which were analysed on a logarithmic (delta CT) scale but presented as fold-changes (anti-logged differences) without standard errors. Comparison of a diet group with the high fat fed group were conducted using t tests. The influence of a single factor and comparisons between the diet groups was tested using one-way ANOVA. Multiple comparisons were tested using either Fisher’s protected or unprotected LSD test. Skewed data was log transformed prior to statistical analysis.

## Electronic supplementary material

Supplementary Information

Supplementary Dataset
