# Supplementary material for: Dietary fibers inhibit obesity in mice, but host responses in the cecum and liver appear unrelated to fiber-specific changes in cecal bacterial taxonomic composition
Source: Sci Rep. 2018 Oct 22;8:15566. doi: 10.1038/s41598-018-34081-8 (PMC6197265; doi:10.1038/s41598-018-34081-8)
Supplement: Supplementary file 1 — Supplementary Information [file 41598_2018_34081_MOESM1_ESM.pdf]

Dietary fibers inhibit obesity in mice, but host responses in the cecum and liver appear unrelated to fiber-specific changes in cecal taxonomic composition

Janice E. Drew[1*], Nicole Reichardt[1,4], Lynda M. Williams[1] Claus-Dieter Mayer[2] Alan W. Walker[1], Andrew J. Farquharson[1], Stavroula Kastora[3], Freda Farquharson[1], Graeme Milligan[4], Douglas J. Morrison[5], Tom Preston[5], Harry J. Flint[1], Petra Louis[1]

[1]The Rowett Institute, [2]Biomathematics and Statistics Scotland, [3]Aberdeen Fungal Group, University of Aberdeen, Foresterhill, Aberdeen AB25 2ZD, UK, [4]Institute of Molecular, Cell and Systems Biology, College of Medical, Veterinary and Life Sciences, University of Glasgow, Glasgow, G12 8QQ, UK, [5]Scottish Universities Environmental Research Centre, University of Glasgow, Rankine Avenue, East Kilbride G75 0QF, UK.

*Corresponding author j.drew@abdn.ac.uk

Supplementary Info Files

Supplementary File S1 Table S1
Experimental diets

Supplementary File S2
Materials and Methods

Supplementary File S3
High resolution image of Figure 3B. [B] Bray Curtis cluster dendrogram of bacterial composition at family level in mice fed mice fed a high fat diet (HFD, red), HFD where 10% of the carbohydrate by weight (5% corn starch, 5% cellulose) was replaced by beta glucan (HFD+bglucan, dark green), apple pectin (HFD+pectin, light green), inulin (HFD+inulin, purple), inulin acetate ester (HFD+inul A, blue), inulin propionate ester (HFD+inul P, light blue), inulin butyrate ester (HFD+inul B, dark blue), inulin propionate and butyrate ester, 5% each (HFD+inul PB, blue-green) and low fat diet (LFD, orange).

Supplementary File S4 Table S2
Illumina sequencing analysis of cecal content. [A] Proportional abundance [%] of each OTU (at 97% identity) subsampled to 12062 reads per sample. The taxonomic classification is shown at the right-hand side. Accession numbers for each sample are given above the sample in each column. Sample codes refer to diet codes as per materials and methods and numbers refer to the individual animal. [B] Lefse analysis of bacterial composition for all individual dietary groups. [C] Lefse analysis of microbiota composition for combined or selected dietary groups. [D] Metastats analysis of microbiota composition for combined or selected dietary groups.

Supplementary File S5 Figure S1Supplementary File S6 Figure S2
Bacterial diversity. [A] Shannon index. [B] Inverse Simpson index. Centre lines show the medians and box plot limits indicate the 25th and 75th percentiles as determined by R software; whiskers extend 1.5 times the interquartile range from the 25th and 75th percentiles, individual animals are presented by dots.

Supplementary File S6 Figure S2
Principal component (PCA) of normalised microarray gene expression data from [A] cecum and [B] liver from mice fed high fat diet (▲), low fat diet (▲), or HFD+inulin (▲) or HFD+inul PB (▲) esters. The PCA profiles explain 22.99% and 19.22 % of the variation in the data respectively. The measurements all fall within the Hotelling T2 95% confidence limit. [C] Differentially expressed probes from microarray analysis of cecum and liver of mice fed HFD+inulin, HFD+inul PB and LFD compared to HFD (selected fold cut off 1.5 and significance levels p<0.01). [D] and [E] Venn diagrams showing patterns of differentially regulated probes with 1.5 fold difference with significance P<0.01 in expression in cecum [D] and liver [E].

Supplementary File S7 Figure S3
Venn diagrams showing patterns of known genes with 1.5 fold differences in expression in cecum [A] and [B] and liver [C] and [D] mice fed a high fat diet (HFD) where 10% of the carbohydrate by weight (5% corn starch, 5% cellulose) was replaced by inulin (Inulin), inulin propionate and butyrate ester, 5% each (Inulin PB) and low fat diet (LFD) relative to HFD fed mice with significance P<0.01.
Supplementary File S8 Table S3

Genes showing common transcriptional responses (known genes >1.5 fold differences, $P<0.01$) in cecum and liver to HFD+inulin and HFD+inul PB compared to HFD or LFD. Genes are listed alphabetically.

Supplementary File S8 Table S3

Genes showing common transcriptional responses (known genes $\geq$1.5 fold differences, $P<0.01$) in cecum and liver to HFD+inulin and HFD+inul PB compared to HFD or LFD. Genes are listed alphabetically.

Supplementary File S1 Supplementary Table S1 Experimental diets

Product		D12451		D14020901		D14020902		D14020903		D14020904		D14020905		D14020906		D14020907		D12450B	
		High Fat (HFD)		HFD+b glucan		HFD+pectin		HFD+inulin		HFD+inul A		HFD+inul P		HFD+inul B		HFD+inul PB		Low Fat (LFD)	
	%	gm	kcal	gm	kcal	gm	kcal	gm	kcal	gm	kcal	gm	kcal	gm	kcal	gm	kcal	gm	kcal
Protein		24	20	24	20	24	20	24	20	24	20	24	20	24	20	24	20	19.2	20
Carbohydrate		41	35	36	35	36	35	36	35	36	35	36	35	36	35	36	35	67.3	70
Fat		24	45	24	45	24	45	24	45	24	45	24	45	24	45	24	45	4.3	10
Total			100		100		100		100		100		100		100		100		100
kcal/gm		4.7		4.7		4.7		4.7		4.7		4.7		4.7		4.7		3.85	
Ingredient		gm	kcal	gm	kcal	gm	kcal	gm	kcal	gm	kcal	gm	kcal	gm	kcal	gm	kcal	gm	kcal
Casein, 80 Mesh		200	800	200	800	200	800	200	800	200	800	200	800	200	800	200	800	200	800
L-Cystine		3	12	3	12	3	12	3	12	3	12	3	12	3	12	3	12	3	12
Corn Starch		72.8	291	1.3	5	29.9	120	29.9	120	29.9	120	29.9	120	29.9	120	29.9	120	315	1260
Maltodextrin 10		100	400	100	400	100	400	100	400	100	400	100	400	100	400	100	400	35	140
Sucrose		172.8	691	172.8	691	172.8	691	172.8	691	172.8	691	172.8	691	172.8	691	172.8	691	350	1400
Cellulose*		50	0	7.1	0	7.1	0	7.1	0	7.1	0	7.1	0	7.1	0	7.1	0	50	0
Beta glucan, 75% active		0	0	114.4	286	0	0	0	0	0	0	0	0	0	0	0	0	0	0
Pectin		0	0	0	0	85.8	172	0	0	0	0	0	0	0	0	0	0	0	0
Inulin		0	0	0	0	0	0	85.8	172	0	0	0	0	0	0	0	0	0	0
Inulin ester A		0	0	0	0	0	0	0	0	85.8	172	0	0	0	0	0	0	0	0
Inulin ester P		0	0	0	0	0	0	0	0	0	0	85.8	172	0	0	42.9	86	0	0
Inulin ester B		0	0	0	0	0	0	0	0	0	0	0	0	85.8	172	42.9	86	0	0
Soybean Oil		25	225	25	225	25	225	25	225	25	225	25	225	25	225	25	225	25	225
Lard		177.5	1598	177.5	1598	177.5	1598	177.5	1598	177.5	1598	177.5	1598	177.5	1598	177.5	1598	20	180
Mineral Mix S10026		10	0	10	0	10	0	10	0	10	0	10	0	10	0	10	0	10	0
DiCalcium Phosphate		13	0	13	0	13	0	13	0	13	0	13	0	13	0	13	0	13	0
Calcium Carbonate		5.5	0	5.5	0	5.5	0	5.5	0	5.5	0	5.5	0	5.5	0	5.5	0	5.5	0
Potassium Citrate, 1 H_2O		16.5	0	16.5	0	16.5	0	16.5	0	16.5	0	16.5	0	16.5	0	16.5	0	16.5	0
Vitamin Mix V10001		10	40	10	40	10	40	10	40	10	40	10	40	10	40	10	40	10	40
Choline Bitartrate		2	0	2	0	2	0	2	0	2	0	2	0	2	0	2	0	2	0
FD&C Yellow Dye #5		0	0	0.05	0	0	0	0.025	0	0.025	0	0	0	0.04	0	0.04	0	0.05	0
FD&C Red Dye #40		0.05	0	0	0	0	0	0.025	0	0	0	0.025	0	0	0	0.01	0	0	0
FD&C Blue Dye #1		0	0	0	0	0.05	0	0	0	0.025	0	0.025	0	0.01	0	0	0	0	0
Total		858.15	4057	858.15	4057	858.15	4057	858.15	4057	858.15	4057	858.15	4057	858.15	4057	858.15	4057	1055.5	4057

*http://www.ifcfiber.com/sellSheets/sfood.php

Supplementary File S2

Materials and Methods

Animals and dietary intervention

The animal studies were licensed under the Animal (Scientific Procedures) Act of 1986 and in accordance with the European Directive on the Protection of Animals used for Scientific Purposes 2010/63/E following ARRIVE guidelines and received approval from the Rowett Institute's Ethical Review Committee. Male C57BL/6 mice, 12 weeks of age and 24-25 g in weight (Harlan, Bicester, UK), were acclimatised for 1 week to single housing on grid floors. Mice were randomly assigned to one of nine dietary groups (n=12) and fed, either: **1.** HFD (high fat diet) (45% of energy from fat) (D12451) **2.** LFD (low fat diet) (10% fat by energy) (D12450B), or the HFD where 10% of the carbohydrate by weight (5% corn starch, 5% cellulose) was replaced by the following dietary fibers: **3.** beta glucan (HFD+bglucan) (Glucagel, DKSH, Milan Italy) **4.** pectin (HFD+pectin) (Sigma-Aldrich, Gillingham, UK) **5.** inulin (Beneo Orafti® HP, DKSH, London UK) **6.** inulin acetate ester (HFD+inul A) **7.** inulin propionate ester (HFD+inul P) **8.** inulin butyrate ester (HFD+inul B) **9.** 5% each of inulin propionate and inulin butyrate ester (HFD+inul PB) (details of diets are provided in Supplementary File S1). Inulin SCFA esters were produced as previously described (Polyviou *et al.*, 2016; Chambers *et al.*, 2015) and incorporated into the animal diets at manufacture. All diets were manufactured by Research Diets, NJ, US http://www.researchdiets.com/opensource-diets/stock-diets/dio-series-diets). During the diet intervention food intake was measured 3 times a week and body weight and body composition was measured weekly by MRI (EchoMRI, Houston, TX, USA).

After 8 weeks animals (n = 12 per group) were killed under terminal anaesthesia and exsanguination either via the hepatic portal vein (n = 4 - 8) or cardiac puncture (n = 4 - 8). Plasma was stored at -80°C. Cecal contents were weighed and genomic DNA extracted for estimation of total bacterial abundance and Illumina sequencing (detailed below) Cecum and liver were weighed, frozen and stored at -80°C prior to extraction of total RNA for gene expression analysis (detailed below).

Cecal bacterial analysis

Genomic DNA (gDNA) was extracted from the cecum contents using the FastDNA® SPIN Kit for Soil (MP Biomedicals, Illkirch, France) according to manufacturer's instructions. The DNA concentration was determined using a Qubit®dsDNA BR Assay Kit on a QubitTM 3.0 Fluorimeter (Thermo Fisher Scientific, Renfrew, UK). The total bacterial abundance was estimated by quantitative PCR using universal primers UniF and UniR against the 16S rRNA gene as described before (Vollmer *et al.*, 2017). The data were expressed as log universal 16S rRNA gene copies per total cecum content. The extracted gDNA (1 ul) was used as a template for sequencing using a 2 step protocol for the V3-V4 region of bacterial 16S rRNA genes using the barcoded fusion primers MiSeq- V3/V4 F (5'- TCG TCG GCA GCG TCA GAT GTG TAT AAG AGA CAG CCT ACG GGN GGC WGC AG -3') and MiSeq-V3/V4 R (5'- GTC TCG TGG GCT CGG AGA TGT GTA TAA GAG ACA GGA CTA CHV GGG TAT CTA ATC C -3'). PCR was performed with Kapa HiFi HotStart Ready Mix (Kapa Biosystems, Inc., Wilmington, MA, USA) DNA polymerase and a per-reaction mix of DNA template (1 µl), 0.2 µM primer (Forward and Reverse), 2X Kapa HiFi HotStart Ready Mix (12.5 µl) and molecular biology grade water to a final volume of 25 µl. Amplification of the V3-V4 region was performed with 20 cycles of PCR and following PCR five reactions per sample were pooled and purified using AMPure XP (Beckman Coulter, Brea, CA, USA) with an AMPure XP to sample ratio of 0.8X prior to indexing. The dual index and sequencing adapters were added

with 8 cycles of PCR and the index PCR was performed with Nextera XT v2 Indices (Illumina, San Diego, CA, USA) with a per-reaction mix of AMPure XP purified PCR template (5 µl), Nextera XT v2 indices i7 and i5 (5 µl each), 2X Kapa HiFi HotStart Ready Mix (25 µl), and molecular biology grade water to a final volume of 50 µl. Following the index PCR the reactions were purified using AMPure XP with an AMPure XP to sample ratio of 1.12X. All samples were quantified on the Qubit Fluorometer 2.0 (Thermo Fisher Scientific, Waltham, MA, USA), quality checked on the Agilent 2200 TapeStation (Agilent Technologies, Inc., Santa Clara, CA, USA), and equimolar pooled. Diluted libraries were sequenced on the Illumina MiSeq using a v3 flow cell with 2x300 bp paired end reads. Sequencing data generated during this study are available in the SRA database under SRA accession SRP117745 and is accessible at http://www.ncbi.nlm.nih.gov/sra/SRP117745.

The data obtained from the Illumina sequencing was analysed using the mothur (v. 1.39.0) software platform (Schloss *et al*., 2009), largely following the MiSeq SOP (Kozich *et al*., 2013) using the University of Aberdeen HPC cluster (Maxwell). In brief, forward and reverse Illumina reads were assembled into paired read contigs. Contigs < 330 bp, > 570 bp, containing ambiguous bases, or homopolymeric stretches of longer than 7 bases, were removed. Sequences that passed this QC step were then aligned against the reference SILVA SEED alignment provided at the mothur website (https://www.mothur.org/wiki/Silva_reference_files#Release_123), and then preclustered allowing up to 4 different bases between reads. Chimera checking resulted in the removal of some genuine and highly abundant OTUs (as assessed by analysis using the BLAST algorithm (Altschul *et al*., 1990). Therefore no chimera checking step was performed for the final analysis. Instead, sequences with 10 sequences or less were removed. Sequences were classified against version 10 of the RDP reference database (Cole *et al*., 2014) and all sequences classified as either "Unknown", "Eukaryota", "Chloroplast" or "Mitochondria", were also removed prior to OTU clustering using the OptiClust algorithm (Westcott & Schloss, 2017) in mothur. All samples were then sub-sampled to 12,062 sequences to ensure equal sequencing depth for subsequent comparisons. Good's coverage estimates at this sequence depth ranged from 99.1 to 99.8%. The final dataset contained a total of 1,302,697 sequences, which were clustered into 1314 OTUs (Supplementary File S3). The Shannon Diversity and inverse Simpson indices were calculated to determine the effects of the different diets on bacterial diversity in each sample. Diversity data were plotted using BoxPlotR (Spitzer *et al*., 2014). Community structures were compared between different diet groups using the Bray-Curtis calculator and significance tested using the Parsimony and AMOVA commands in mothur (Schloss *et al*., 2009). Metastats (White *et al*., 2009) and LEfSE (Segata *et al*., 2011) were used in mothur to test for significant differences overall in microbiota composition at the OTU, genus, family and phylum levels between the combined fibre-added and HFD/LFD groups. The Benjamini-Hochberg method was used to account for multiple comparisons when using Metastats (Benjamini and Hochberg, 1995). LEfSe analysis (Segata *et al*., 2011) was also conducted in mothur to assess whether or not specific bacterial taxa were associated with the different diets. Cluster dendrograms were created using the tree.shared command in mothur with the Bray-Curtis calculator, and visualized using iTOL (Letunic and Bork, 2016).

Whole Genome Microarray Analysis

RNA was extracted from 20 mg liver and cecum (n = 6) using an RNeasy Mini Kit (Qiagen, Crawley, UK) incorporating DNase digestion, followed by quantification using a NanoDrop Spectrophotometer (NanoDrop Technologies). Quality was assessed using an Agilent

Bioanalyser (Agilent Technologies). Total RNA extracted from liver and cecum was microarrayed with SurePrint G3 Mouse GE 8x60K Microarray G4852A (Agilent Technologies, UK) using the manufacturer's protocol. The microarrays contained 60-mer probes with 39,430 Entrez gene RNAs and 16,251 lincRNAs represented. Cy3 labelled cRNA was hybridized to each array rotating at 65°C for 17 h. After washing, the arrays were scanned using a SureScan High Resolution Scanner (Agilent Technologies). The statistical programming language R (v3.0.2) was used to analyse the raw expression data. Gene expression values were analysed on log2 scale. A cyclic loess normalisation was used to normalise the one-channel data across arrays (Bolstad *et al*., 2003). Values of spots that were represented by identical oligos were averaged across duplicates. The quality control reports from the Agilent system and explorative statistical analysis (principal component analysis, hierarchical clustering) were used to check for data quality and one array from the cecum HFD+inulin propionate/butyrate ester group was eliminated as it appeared a clear outlier in the explorative statistics and the Agilent output showed large areas of smear on the slide. Technical issues led to elimination of one of the cecum LFD mouse samples from the microarray analysis. Principal Component Analysis (PCA) was performed on the resulting data using SIMCA-P+ 12.0 software (MKS Instruments UK Ltd, Cheshire). The Linear Models for Microarray Data (LIMMA) package was used to detect the significant expression changes in probe sets in comparison with the HFD mice (Ritchie *et al*., 2015). Log2 ratios of treatment effects were back transformed to obtain expression relative to the mice fed a HFD. The data discussed in this publication have been deposited in NCBI's Gene Expression Omnibus (Edgar *et al*., 2002) and are accessible through GEO Series accession number GSE106375 (www.ncbi.nlm.nih.gov/geo/query/acc.cgi?acc=GSE106375).

Gene Ontology (GO) term analysis and network construction were performed on differentially expressed probe sets (see section above) filtered to extract all the known genes that met the criteria of $\geq$1.5 fold change at $p < 0.01$. Gene list comparisons were then assessed using Venny (Oliveros). Functional enrichment analysis and network construction was conducted using Cytoscape v3.4 and the CluGO plug-in (Shannon *et al*., 2003, Bindea *et al*., 2009).

Confirmation of microarray identified gene changes using custom designed RT Profiler PCR arrays

Genes showing altered responses to HFD+inulin or HFD+inulin propionate and butyrate esters in cecum were identified from microarray analysis and validated using a custom designed RT Profiler PCR Array (Qiagen). Cecal tissue (50 mg) from the experimental mice was provided to QIAGEN for total RNA extraction (n=6). RT Profiler PCR Array provided a means of simultaneously comparing expression of the selected gene targets in response to all of the dietary interventions. Genomic DNA (GDC), positive PCR (PPC) and reverse transcription (RTC) controls were incorporated for each extracted sample of total RNA. *UBE2D2* was included in the RT Profiler PCR Array analysis for normalisation as the current microarray analysis (cecum) and previous studies indicated stable expression of *UBE2D2* in mouse colon (Drew *et al*., 2016) tissues. The custom designed RT Profiler PCR Array contained primer sets in duplicate for each gene target, optimised for SYBR green real-time RT-PCR detection using a 384-well plate format. The raw threshold data cycle number (C_t) generated by PCR was delivered by Qiagen and used to calculate transcript levels relative to each of the reference genes, *UBE2D2* (ΔC_t). Subsequent fold expression changes between experimental groups relative to *UBE2D2* were calculated from the $\Delta\Delta C_t$ values.

Real-time PCR

Complementary cDNA templates for real-time PCR assays were prepared from Superscript II (Invitrogen) reverse transcribed total RNA (2ug) (n = 6). Taqman real-time PCR assays were performed using VIC and FAM reporter dyes. All reactions were carried out using 1x dilution of Taqman Fast Universal PCR 2x mastermix No AmpErase® Ung (Applied Biosystems, UK) according to the manufacturer's instructions. Taqman primer assays for CNR1 (Mm00432621_s1 FAM-labelled), *Enho* (Mm01223541_m1 FAM-labelled), *SAA1* (Mm00656927_g1), *SAA1* (Mm04208126_mH) and *UBE2D2* (Mm00785931_s1 VIC-labelled), were supplied by Applied Biosystems. Duplicate samples were PCR assayed using the ABI-7500Fast (Applied Biosystems, UK), with a FAM-labelled target gene and VIC-labelled reference gene (*UBE2D2*, Mm00785931_s1). A two-step cycling programme of 95ºC 20 seconds, then 40 cycles of 95ºC for 3 sec and 60ºC for 30 sec was used. The threshold cycle number (C_t) was measured using the ABI7500Fast associated software (Applied Biosystems, UK). Transcript levels relative to the reference gene, *UBE2D2*, were calculated (ΔC_t). Fold expression changes between experimental groups relative to *UBE2D2* were calculated from the $\Delta\Delta C_t$ values.

Liver Adropin (Enho)

Liver soluble protein homogenates were prepared by bead-grinding 20 mg tissue using a PreCellys 24 machine (Bertin Technologies, UK) with 400 µl of phosphate buffer (pH7.4). Homogenates were centrifuged at 5000 x *g* and aliquots of supernatants stored at -70°C until analysis. An enzyme-linked immunosorbent assay (ELISA) kit (Cusabio USA) was used to determine changes in adropin protein concentrations in accordance with manufacturer's instructions. Adropin concentrations were standardised as mg total soluble protein content per mg liver tissue.

References

1. Altschul SF, Gish W, Miller W, Myers EW, Lipman DJ. Basic local alignment search tool. *J Mol Biol* 1990; 215: 403-410.
2. Benjamini Y, Hochberg Y. Controlling the False Discovery Rate: A Practical and Powerful Approach to Multiple Testing. *J Royal Stat Soc Series B* 1995; 57: 289-300.
3. Bindea G, Mlecnik B, Hackl H, Charoentong P, Tosolini M, Kirilovsky A, *et al.*. ClueGO: a Cytoscape plug-in to decipher functionally grouped gene ontology and pathway annotation networks. *Bioinformatics* 2009: 25: 1091-3.
4. Bolstad BM, Irizarry RA, Astrand M, Speed TPA. Comparison of normalization methods for high density oligonucleotide array data based on bias and variance. *Bioinformatics* 2003; 19: 185-193.
5. Cole JR, Wang Q, Fish JA, Chai B, McGarrell DM, Sun Y, *et al.* (2014) Ribosomal Database Project: Data and tools for high throughput rRNA analysis. *Nucl Acids Res* 42: D633-D642.
6. Drew JE, Farquharson AJ, Horgan GW, Williams LM. Regulation of sirtuin and nicotinamide adenine dinucleotide biosynthetic pathways in high-fat fed C57Bl/6 mice. *J Nutr Biochem* 2016; 37: 20-29.
7. Edgar R, Domrachev M, Lash AE. Gene Expression Omnibus: NCBI gene expression and hybridization array data repository. *Nucl Acids Res* 2002; 30:207-210.

8. Kozich J, Westcott SL, Baxter NT, Highlander SK, Schloss PD Development of a dual-index sequencing strategy and curation pipeline for analysing amplicon sequence data on the miseq illumina sequencing platform. *Appl Environ Microbiol* 2013; 79: 5112–5120.
9. Letunic I, Bork P. Interactive tree of life (iTOL) v3: an online tool for the display and annotation of phylogenetic and other trees. *Nucleic Acids Res* 2016; 44: W242-245.
10. Oliveros JC. (2007-2015) Venny. *An interactive tool for comparing lists with Venn's diagrams.* http://bioinfogp.cnb.csic.es/tools/venny/index.html
11. Ritchie ME, Phipson B, Wu D, Hu Y, Law CW, Shi W *et al.*. Limma powers differential expression analyses for RNA-sequencing and microarray studies. *Nucl Acids Res* 2015; 43: e47.
12. Schloss PD, Westcott SL, Ryabin T, Hall JR, Hartmann M, Hollister EB, *et al.*. Introducing mothur: open-source, platform-independent, community supported software for describing and comparing microbial communities. *Appl Environ Microbiol* 2009; 75: 7537–7541.
13. Segata N, Izard J, Waldron L, Gevers D, Mirpolsky L, Garrett WS, *et al.*. Metagenomic biomarker discovery and explanation. *Genome Biol* 2011; 12: R60.
14. Shah C, Hari-Dass R, Raynes JG. Serum amyloid A is an innate immune opsonin for Gram-negative bacteria. *Blood* 2006; 108: 1751-1757.
15. Shannon P, Markiel A, Ozier O, Baliga NS, Wang JT, *et al.* Cytoscape: a software environment for integrated models of biomolecular interaction networks. Genome Res. 2003; 13:2498-504.
16. Spitzer M, Wildenheim J, Rappsilber J, Tyers M. BoxPlotR: a web tool for generation of boxplots. *Nat Methods* 2014; 11: 121–122.
17. Vollmer M, Schröter D, Esder S, Farquharson FM, Neugart S, Duncan S, *et al*. Chlorogenic acid versus amaranth's caffeoylisocitric acid - Gut microbial degradation of caffeic acid derivatives *Food Res Int* 2017; 100:375– 384.
18. Westcott SL, Schloss PD. OptiClust, an improved method for assigning amplicon-based sequence data to operational taxonomic units. *mSphere* 2017; 2: e00073-17.
19. White, J.R., Nagarajan, N., and Pop, M. (2009) Statistical methods for detecting differentially abundant features in clinical metagenomic samples. PLoS Comput Biol 5: e1000352.

Supplementary File S3

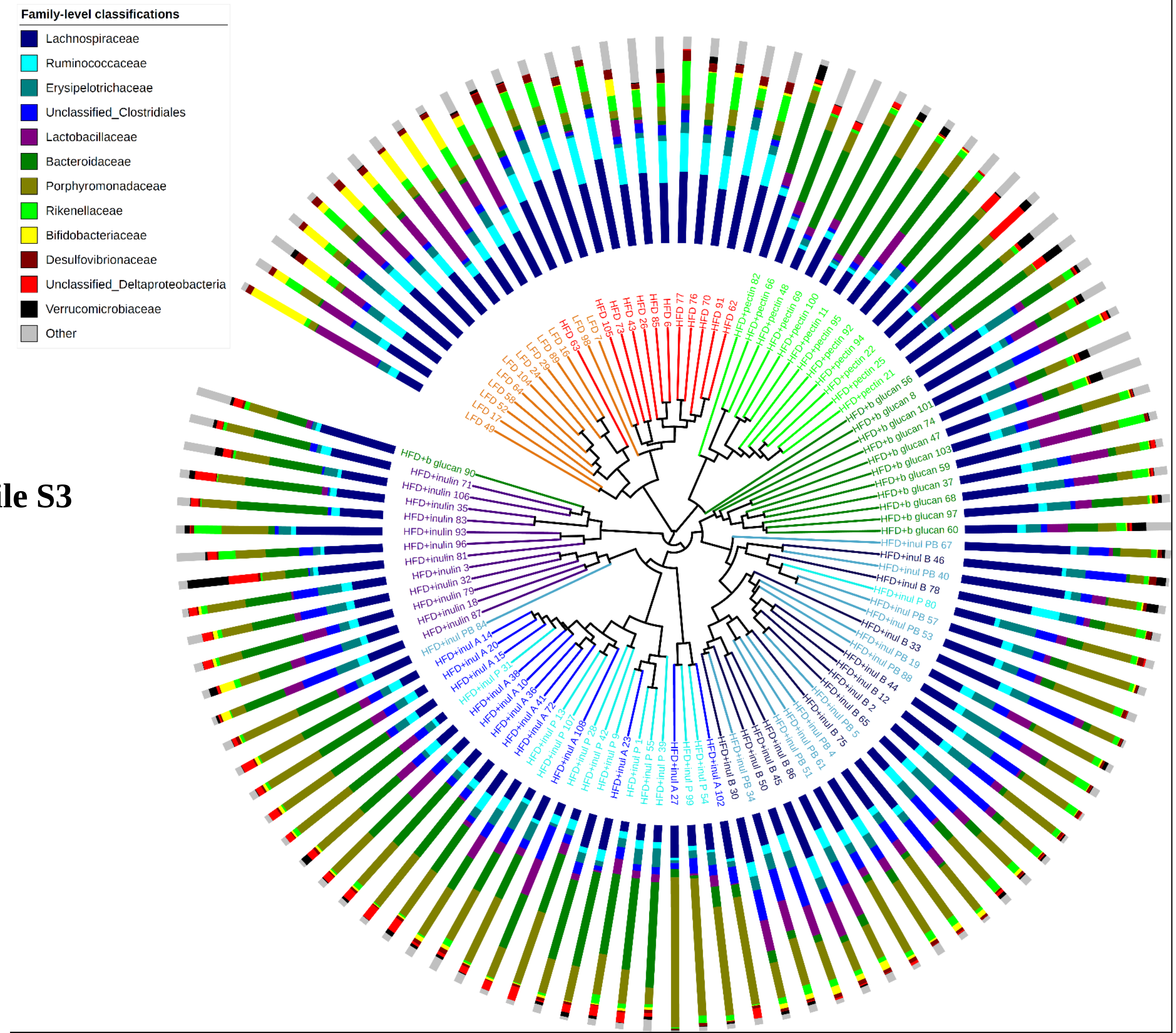

Supplementary File S5 Figure S1Supplementary File S6 Figure S2

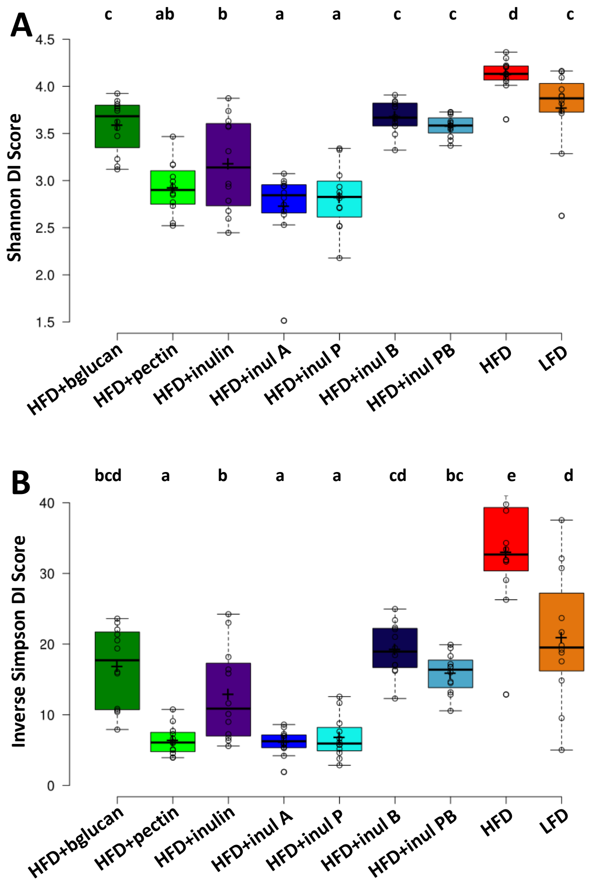

Supplementary File S6 Figure S2

[A] [B]

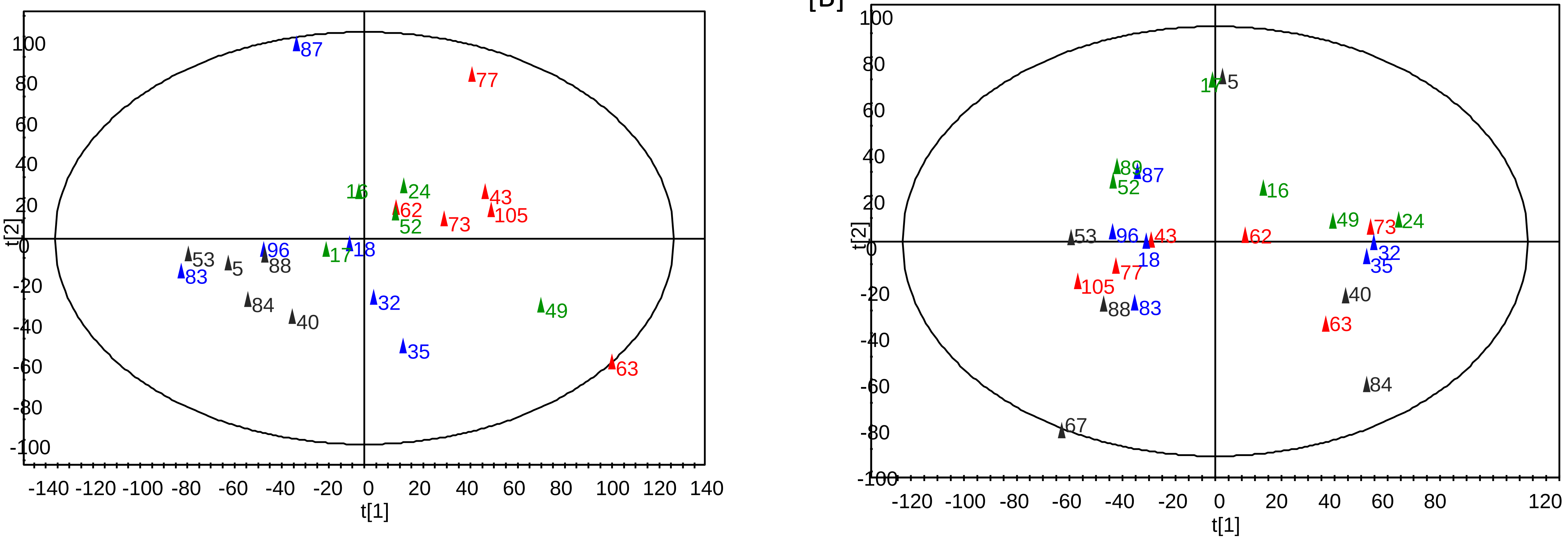

[C]

Tissue	HFD+inulin	HFD+inul PB	LFD
Cecum	741	1614	151
Up-regulated	364	911	69
Down-regulated	377	703	82
Liver	68	53	196
Up-regulated	30	30	107
Down-regulated	38	23	89

[D] [E]

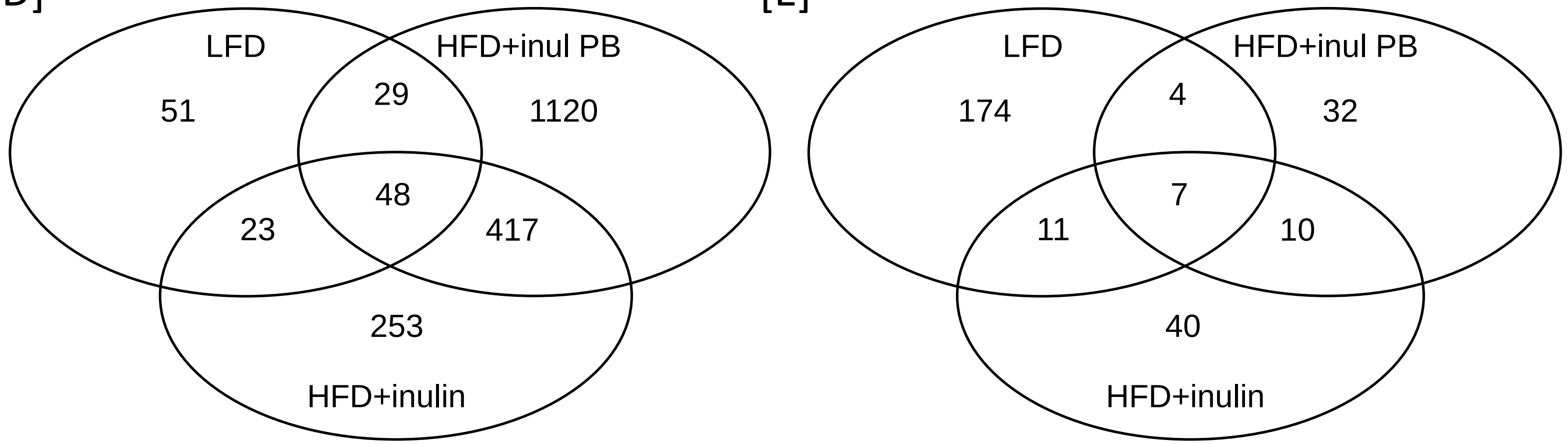

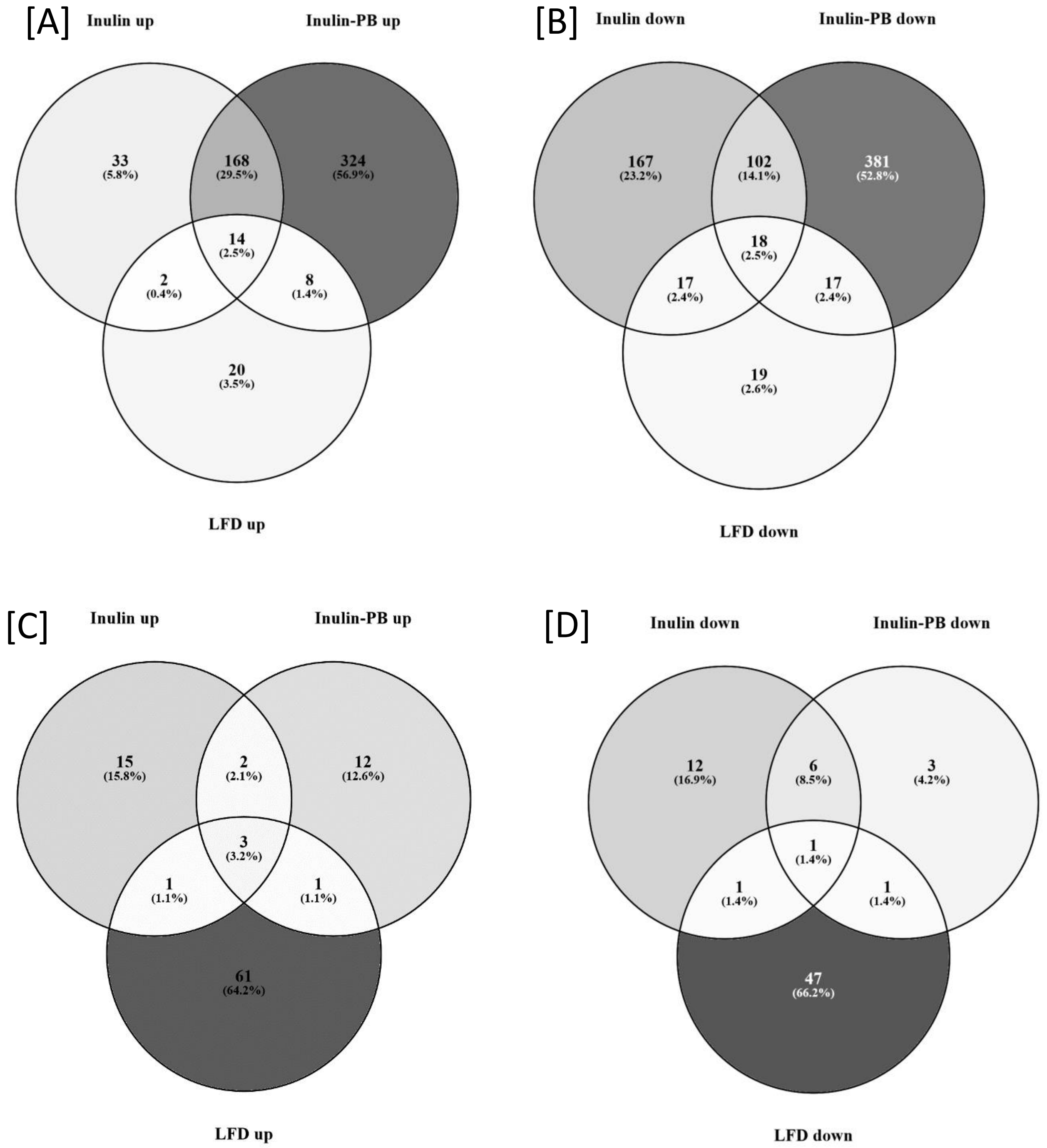

Supplementary File S7 Figure S3

Supplementary File S8 Table S3

Genes showing common transcriptional responses to HFD+inulin and HFD+inul PB compared to HFD or LFD

Cecum		Liver	
**Down regulated**	**Up regulated**	**Down regulated**	**Up regulated**
Abcc6	Aagab	Cyp21a1	Cox7a1
Acsm3	Acss1	Itgax	Enho
Adam18	Adora2b	Lcn2	
Agr2	Ahnak	Rarres1	
Amph	Ak4	Saa1	
Anxa5	Akap13	Saa2	
Atp2c2	Akr1c14		
B3galt5	Aldh4a1		
Bcas1	Ano9		
Bex2	Arrdc4		
C2cd4b	Asb4		
Capn9	Atg16l2		
Ccl9	Bmp2		
Ces1b	Cadm4		
Chst4	Camk2n1		
Cldn4	Car4		
Cldn5	Carhsp1		
Creb3l3	Casp3		
Creb3l4	Casr		
Cyp2c67	Ccdc116		
Cyp2c68	Ccrn4l		
Cyp4f14	Cd84		
Cyp7b1	Cdc14a		
Dmp1	Ces1f		
Doc2b	Clec2e		
Dusp26	Cpeb4		
Edil3	Cspg5		
Fabp7	Cxadr		
Fer1l4	Cyp2d10		
Fgf7	Cyp2d11		
Fut4	Cyp2d12		
Gem	Cyp2d34		
Gfra3	Cyp2d9		
Gjb4	Daglb		
Gnl3	Dio1		
Gprin1	Dsg3		
Gsta2	Duoxa2		
Gsta3	Emc9		
Gsta4	Emp2		
Hsd17b14	Evpl		
Id4	Exd1		
Il1rl1	Fads3		
Il7r	Fam3c		
Inmt	Fam82b		
Insig1	Fbxo10		

Krt7	Fbxo25
Lama3	Fcho1
Lef1	Fgfr2
Lrrc26	Flywch2
Ltbp2	Gdpd2
Lypd6b	Ggh
Me1	Glrx
Meg3	Gnal
Mest	Gnb5
Mfsd4	Gpr17
Mmp11	Gstm6
Muc16	H2-Bl
Muc2	H2-T3
Mxra8	Hcrtr1
Myl1	Helz2
Nr1h3	Hist1h1c
Ntrk3	Hist1h2bq
Olfr517	Hist1h4m
Omg	Hsd3b2
Otc	Ier5
Oxct1	Ifnar1
Pcsk9	Igf2bp2
Pdha1	Ikzf2
Pfkfb4	Inpp5j
Phgdh	Iqsec2
Prss12	Junb
Rab27b	Kazn
Rab30	Kctd17
Ramp1	Kif21b
Rap1gap	Kifc2
Rbp7	Kremen2
Reg4	Krt12
Rnf186	Krtap3-1
Sema7a	Lrrtm1
Sfn	Ly6a
Slc12a8	Ly6f
Slc40a1	Ly6g
Slc41a2	Mal
Slc4a11	Maoa
Slc51b	Mapk13
Slc7a7	Mboat2
Slc9a3	Meis3
Sncg	Mertk
Snn	Mettl7b
Spdef	Mid1
Spink4	Mier1
Sval1	Mov10
Sybu	Mpp6
Syde2	Muc4
Tarsl2	Mycbpap

Tgm5	Nacad
Tln2	Nceh1
Tmem117	Nfatc2
Tmem184c	Nfkbiz
Tpsg1	Odf3b
Tub	Olfr487
Wnt10a	Oma1
	Papss2
	Pde6a
	Pde8a
	Pitpnm3
	Pitx2
	Pla2g15
	Pla2g2c
	Pla2g3
	Plekha3
	Plekhs1
	Pnpo
	Ppap2a
	Prelid2
	Prss30
	Ptp4a1
	Rdh16
	Rdh18-ps
	Rec8
	Rgl3
	Rnf152
	Rnf19b
	Rph3al
	Saa1
	Saa2
	Sat2
	Sdr42e1
	Sectm1a
	Sectm1b
	Selenbp1
	Selm
	Sesn2
	Sh3bp1
	Slc13a2
	Slc20a1
	Slc25a34
	Slc26a3
	Slc27a2
	Slc35d1
	Slc37a2
	Slc4a7
	Slc5a8
	Slco2a1
	Spaca4

Speer4d
Speer5-ps1
St3gal5
Stard5
Tat
Tex19.1
Tgoln2
Tm4sf20
Tmc1
Tmc5
Tmem189
Tmem220
Tmem35
Tmprss12
Tmprss4
Tnfrsf11a
Tnfrsf21
Tnni1
Ttc39c
Tubb2a
Ypel3
Zfand5
Zfp820
